# Targeting Aberrant Expression of STAT3 and AP-1 Oncogenic Transcription Factors and HPV Oncoproteins in Cervical Cancer by *Berberis aquifolium*

**DOI:** 10.3389/fphar.2021.757414

**Published:** 2021-10-28

**Authors:** Tejveer Singh, Arun Chhokar, Kulbhushan Thakur, Nikita Aggarwal, Pragya Pragya, Joni Yadav, Tanya Tripathi, Mohit Jadli, Anjali Bhat, Pankaj Gupta, Anil Khurana, Alok Chandra Bharti

**Affiliations:** ^1^ Molecular Oncology Laboratory, Department of Zoology, University of Delhi (North Campus), Delhi, India; ^2^ Dr. DP. Rastogi Central Research Institute of Homeopathy, Noida, India; ^3^ Central Council for Research in Homeopathy, New Delhi, India

**Keywords:** Cervical cancer, Berberis aquifolium, Cell cycle, STAT3, AP-1, Oncoprotein, HPV16 E6, Molecular Docking

## Abstract

**Background:** Present study examines phytochemical preparation that uses berberine’s plant source *B. aquifolium* root for availability of similar anti-cervical cancer (CaCx) and anti-HPV activities to facilitate repurposing of the *B. aquifolium* based drug in the treatment of CaCx.

**Purpose:** To evaluate therapeutic potential of different concentrations of ethanolic extract of *B. aquifolium root* mother tincture (BAMT) against HPV-positive (HPV16: SiHa, HPV18: HeLa) and HPV-negative (C33a) CaCx cell lines at molecular oncogenic level.

**Materials and Methods:** BAMT was screened for anti-proliferative activity by MTT assay. Cell cycle progression was analyzed by flowcytometry. Then, the expression level of STAT3, AP-1, HPV E6 and E7 was detected by immunoblotting, whereas nuclear localization was observed by fluorescence microscopy. Phytochemicals reportedly available in BAMT were examined for their inhibitory action on HPV16 E6 by *in silico* molecular docking.

**Results:** BAMT induced a dose-dependent decline in CaCx cell viability in all cell types tested. Flowcytometric evaluation of BAMT-treated cells showed a small but specific cell growth arrest in G1-phase. BAMT-treatment resulted in reduced protein expression of key transcription factors, STAT3 with a decline of its active form pSTAT3 (Y705); and components of AP-1 complex, JunB and c-Jun. Immunocytochemistry revealed that BAMT did not prevent the entry of remnant active transcription factor to the nucleus, but loss of overall transcription factor activity resulted in reduced availability of transcription factors in the cancer cells. These changes were accompanied by gradual loss of HPV E6 and E7 protein in BAMT-treated HPV-positive cells. Molecular docking of reported active phytochemicals in *B. aquifolium* root was performed, which indicated a potential interference of HPV16 E6’s interaction with pivotal cellular targets p53, E6AP or both by constituent phytochemicals. Among these, berberine, palmatine and magnoflorine showed highest E6 inhibitory potential.

**Conclusion:** Overall, BAMT showed multi-pronged therapeutic potential against HPV infection and cervical cancer and the study described the underlying molecular mechanism of its action.

## Introduction

*Berberis aquifolium* commonly known as Oregon grape or Mahonia is a widely used medicinal plant by native North American Indian tribes, in Chinese medicine and in many other traditional medicines (Duke and Ayensu 1985; Satyavathi et al., 1987; Moerman 1998). The root and root bark is used as laxative, diuretic, cholagogue, alterative, and blood tonic (Bown and Herb Society of America. 1995; Chevallier 1996). *B. aquifolium* root extracts are used to treat psoriasis, syphilis, haemorrhages, gastric disorders, sore throats, and bloodshot eyes. *B. aquifolium* is extensively used in alternative medicine system (Boericke 2001). Mother tincture prepared from *B. aquifolium* root is a remedy for the skin, chronic catarrhal affections, secondary syphilis, hepatic torpor, and lassitude.

Briefly, CaCx is the fourth leading cancer of women with an annual incidence of 570,000 and mortality 311,000 (Arbyn et al., 2020). To make things worse, over 85% CaCx cases and resultant deaths are reported from low resource countries. Contrastingly, China and India alone collectively contributed more than a third of the global burden. CaCx is caused by persistent infection of high-risk HPV primarily HPV16 (> 50% cases) and followed by HPV18 (∼15% cases) (IARC 2012). HPV codes for two key oncoproteins E6 and E7 that drive cervical carcinogenesis by physically interacting and targeting functions of p53 and pRB, respectively (Yeo-Teh et al., 2018). Loss of p53 and pRb are both key players in cell cycle dysregulation. Among these two, E6 plays a more dominant role by making a complex with p53 in association with host-derived E6-associated protein (E6AP) (Scheffner et al., 1990; Huibregtse et al., 1991). 3D-structure of co-crystallized E6-E6AP-p53 revealed binding of E6 to the conserved domain of E6AP occurs *via* the LxxLL consensus sequence (Martinez-Zapien et al., 2016). E6 and E6AP together form a heterodimer that degrades p53.

Expression of E6/E7 is tightly controlled by early promoter located downstream to an untranslated Upstream Regulatory Region (URR) that contains *cis*-regulatory elements for binding of viral regulatory protein E2 and host-derived transcription factors like AP-1, STAT3, among several others (Shishodia et al., 2018). AP-1 is a redox sensitive transcription factor (Angel et al., 1987), whereas STAT3 is a pro-carcinogenic and pro-inflammatory transcription factor (Bromberg et al., 1999). Both the transcription factors are found overexpressed and constitutively active in the cervical cancer cells and their expression and activity increased with disease severity (Prusty and Das 2005; Shukla et al., 2010). These transcription factors played a functional regulatory role in the expression of viral oncogenes (Shukla et al., 2013). Experimental targeting of E6/E7 activity/expression by targeting viral transcription using synthetic pyrrolidine-dithiocarbamate (PDTC) (Rosl et al., 1997), or natural anti-oxidants (curcumin, berberine or other phytochemicals), anti-sense oligos, RNA interference or by different genome targeting approaches induced remarkable growth inhibition, cell cycle arrest and induction of apoptosis/senescence in CaCx cells (reviewed in (Bhartih et al., 2018)).

Despite being a highly preventable disease due to viral etiology, availability of prophylactic vaccines and substantial understanding of the disease mechanism leading to several preclinical leads (Bharti et al., 2018), there is no clinically-available anti-HPV therapeutics as yet. Therefore, taking leads obtained from berberine, we explored preparations in clinical practice that may potentially contain berberine as one of the constituent. In this regard, *B. aquifolium* mother tincture (BAMT) which is an ethanolic extract of *B. aquifolium* root, was studied in the current investigation. Apart from berberine, *B. aquifolium* root contains several other medicinally-useful constituents like berbamine, hydrastine, jatrorrhizine, magnoflorine, oxyacanthine, columbamine, obamegine, aromoline, and palmatine (USDA 1992-2016) that can potentially act against HPV and/or CaCx. Therefore, in the present investigation, we examined if phytomedicine based on *B. aquifolium* could be repurposed for treatment of CaCx by using well-established cell and molecular biology and bioinformatics assays. We evaluated cell viability, cell cycle, effects on transcription regulators of viral oncogenes, and expression of oncoprotein E6 and E7. Further, we examined the molecular interaction of constituent phytochemicals *in silico* on HPV16 E6 and its ability to interact with corresponding cellular targets that promote cervical carcinogenesis.

## Materials and Methods

### Chemical and Reagent

*B. aquifolium* MT (ethanol content 70% in water; batch no.–UM180120 (3/2018)) were procured from authoritative sources SBL–Sharda Boiron Laboratories Private Limited, India; a GMP-certified firm. The alcohol (ethanol) utilized to prepare the drug was used at corresponding concentration uniformly as negative control. The cell culture media, trypsin-EDTA and penicillin-streptomycin solution were procured from HiMedia (India). MTT (3-(4, 5-Dimethylthiazol-2-yl)-2, 5-diphenyltetrazolium bromide), DAPI (4′,6-diamidino-2-phenylindole) and all other reagents were of analytical grades and were procured from Sigma-Aldrich Chemicals unless specified.

### Cell Culture

Human CaCx cell lines with known HPV positivity for HPV type 16 - SiHa and HPV type 18 - HeLa; and HPV negative C33a were originally procured from ATCC and were maintained in DMEM/MEM supplemented with 10% FBS and 1% antibiotic (penicillin and streptomycin) as *mycoplasma* contamination-free cultures. All cell lines were maintained in 5% CO_2_ incubator at 37°C. The cell lines were tested for HPV positivity and genotype by using HPV consensus L1 and HPV type-specific PCR periodically for ensuring authenticity and rule out cross contamination (Supplementary Figure SF1).

### MTT Cell Viability Assay

Cell viability assay was performed as described earlier (Bharti et al., 2003) with minor modifications. Briefly, CaCx cells (2 × 10^3^) were seeded in triplicate in a tissue culture grade 96-well plate in a final volume of 0.1 ml overnight at 37°C in a CO_2_ incubator and subjected to drug treatment as indicated by diluting in complete medium (10–0.01%). Ethanol at corresponding strength was used uniformly as vehicle control to treat CaCx cells. Thereafter, 0.025 ml of MTT (3-(4, 5-dimethylthiazol-2-yl)-2, 5-diphenyltetrazolium bromide) solution (5 mg/ml in PBS) was added to each well. After 2 h incubation at 37°C, the culture medium was removed and 100 µl lysis buffer (20% SDS; 50% dimethylformamide) was added and incubated overnight at 37°C for solubilisation of formazan crystals. The optical density (OD) at 570 nm was measured using a 96-well Multiscanner Autoreader (Biotek, United States). BAMT was inherently colored and hence, interfered with colorimetric assays at higher concentrations. Therefore, cell-free reagent controls were uniformly used to subtract reagent OD. The percentage of viable cells was calculated using the following formula:

Percent Cell Viability=(OD of the experiment samples/OD of the control)×100.

*Calculation of IC*_*50*_ *of the homoeopathic preparations:* IC_50_ of the drug was calculated with non-linear regression analysis on log transformed normalized values of BAMT dosage points using Prism (Version 8.0.2; GraphPad Software, CA, United States).

### Cell Cycle Analysis by Flowcytometry

Cell cycle analysis was performed by RNase and propidium iodide (PI) staining followed by flowcytometry as described earlier (Bharti et al., 2003) with some minor modifications. Briefly, CaCx cells were seeded into 6-well plates at a density of 5 × 10^4^ cells/well. Cells were treated with indicated concentrations of BAMT or vehicle control. At completion of treatment, the cells were harvested by trypsinization using 0.3 ml of 1X Trypsin-EDTA solution, washed with PBS containing 10% serum, fixed in 70% ethanol. After overnight incubation at −20°C, cells were washed and stained with PI by suspending in staining buffer (10 μg/ml (BD Biosciences, United States), 0.5% Tween-20; 0.1% RNAase) for 40 min at 4°C in dark. The cells were analysed by FACSAriaIII equipped with FACSDiva software (BD Biosciences). A total of 10,000 events were acquired for each sample. For analysis, the cells were gated to exclude cell debris, cell doublets and cell clumps to identify the single cell population first using PI width vs. PI area. The gates were applied to the PI histogram plot.

### Immunoblotting

Levels of different proteins were evaluated by immunoblotting as described earlier (Shukla et al., 2010). Briefly, CaCx cells (1 × 10^6^ cells/100 mm plate) were treated as described. At the end of treatment, the cells were scraped, collected and washed with ice cold 1X PBS. Total cellular proteins were extracted by re-suspending the pellet in the cell lysis buffer (20 mM Tris (pH 7.4), 250 mM NaCl, 2 mM EDTA (pH 8.0), 0.1% Triton X-100, 0.01 mg/ml aprotinin, 0.005 mg/ml leupeptin, 0.4 mM PMSF, and 4 mM Na_3_VO_4_). Lysates were spun at 14,000 rpm in a microfuge for 10 min to remove insoluble material and clear supernatant for each sample was collected. The concentration of proteins was determined by spectrophotometric method and the proteins were stored in aliquots at −80°C till further use. Proteins (40 μg/lane) were resolved in 10–12% polyacrylamide gel using 2X Laemmli buffer (100 mM Tris-HCL pH 8.0, 20 mM EDTA pH 8.0, 4% SDS, 20% glycerol, 10% β-mercaptoethanol, 0.02% bromophenol blue) and transferred to PVDF membranes (0.22 µM; Millipore Corp, Bedford, MA, United States) by wet transfer method. During PAGE, border lanes were loaded with Precision Plus Protein Dual Color Standards (Bio-Rad, United States; Catalog# 161–0374) protein pre-stained marker. Blot was cut between 25 and 37 kDa bands. Upper blot (with ≥37 kDa proteins) was used for detection of transcription factors and lower blot (with ≤25 kDa proteins) was used for detection of HPVE6/E7 proteins. The membranes were blocked in 5% BSA (prepared in 0.1% Tween-20 in Tris-Borate Saline) and probed with specific antibodies in a probing-stripping-reprobing cycle**.** Upper blot was first incubated overnight at 4°C in the pre-standardized dilution of primary antibody against pSTAT3(Y705), and subsequently re-probed with anti-STAT3, anti-JunB, anti-c-Jun, and β-actin. Similarly, lower blot was incubated first with HPV16/18 E6 and subsequently re-probed with either HPV16 E7 or HPV18 E7 depending upon the cell type. Antibodies and their specific dilution in the blocking solution used in different assays are described in Supplementary Table S1. Absence of leftover signal following stripping was ascertained before the next reprobing cycle. During each probing, blots were washed, incubated with horse reddish peroxidase (HRP)-conjugated anti-mouse/rabbit IgG secondary antibodies and visualized by Luminol detection kit (Santa Cruz Biotech, United States) under Amersham Imager 600 (GE Life Sciences ABI, Sweden). The western blot membranes were stripped at each interval using mild stripping buffer (1.5% glycine, 0.1% SDS, 1% Tween-20 pH-2.2) for 15 min at room temperature followed by re-blocking. β-actin expression was used as an internal control. The quantitative densitometric analysis of the bands was performed using ImageJ software (NIH, United States).

### Immunocytochemistry and Fluorescence Microscopy

Subcellular localization of various proteins was determined by ICC as described earlier (Bharti et al., 2003) with minor modifications. CaCx cells were seeded on coverslips in 6 well plates at a density of 5,000 cells/well. After overnight incubation, the cells were treated. When the treatment got over, the medium was removed, and cells were rinsed with 1X PBS for 3 times. Cells were fixed in 4% paraformaldehyde for 20 min and permeabilized with 0.2% Triton X-100 in 1X PBS. Cells were blocked with 5% BSA in 1X PBS for 1 h. Cells were incubated with primary antibodies (Supplementary Table ST1) for 3 h followed by incubation with fluorescently tagged secondary antibodies for 1 h. Counter staining was done with DAPI at a concentration of 50 ng/ml. Finally, the coverslips were mounted on a microscope slide with Fluor mount as mounting media. Preparations were visualized using a ZEISS ImagerZ2 microscope. Fluorescence intensity analyses were performed using ImageJ software (U.S. National Institutes of Health).

### *In Silico* Molecular Docking of Phytochemicals to HPV16 E6 Molecule

#### Preparation of HPV16 E6 Structures

Two recent protein structures of HPV16 E6 i.e. chain B of 6SJA (aa7-158; resolution 1.5 Å) that represents native conformation of E6 (non-p53 interacting) (Suarez et al. 2019) and chain F of 4XR8 (aa8-158; resolution 2.25 Å) that represents p53-interacting conformation of E6 (Martinez-Zapien et al. 2016) were downloaded from RCSB PDB website (http://www.rcsb.org/pdb/home/home.do). The analysable part of Chain B (aa7-aa140) and chain F (aa7-aa143) corresponding to aa14 -aa147 and aa14 -aa150, respectively in UniProtKB Reference sequence for E6 P03126 were used for analysis. Chain B of 6SJA differed from reference protein at position 54 as it had ARG instead of PHE. The original crystal structures were processed in PyMOL to remove non-E6 components of the structure and chain B of 6SJA and chain F of 4XR8 structure were isolated. Chain B of 6SJA and chain F of 4XR8 structure were independently used to dock phytochemical ligands. Apart from RCSB PDB database, Online file format converting tools (https://cactus.nci.nih.gov/translate/), NCBI (https://www.ncbi.nlm.nih.gov/), Online Alignment Tool (http://www.ebi.ac.uk/Tools/psa/emboss_needle/) and softwares AutoDock Tools 1.5.6 (ADT) (http://autodock.scripps.edu/resources/adt), PyMOL (https://www.PyMOL.org/) and Discovery Studio (http://accelrys.com/products/collaborative-science/biovia-discoverystudio/visualization-download.php) were used in present study. The protein structures were processed before docking by ADT. The water molecules were deleted, non-polar hydrogens added into carbon atoms of the E6 molecule and Kollman charges were also assigned. Gasteiger charges were assigned and torsions degrees of freedom were allocated by ADT.

#### Preparation of Ligands

The phytochemicals likely to be present in the root of *Berberis aquifolium* were identified from Dr. Duke’s Phytochemical and Ethnobotanical database (USDA 1992–2016). The molecular structures of the phytochemicals were retrieved from NCBI PubChem and converted to. pdb format using Online SMILES Translator and Structure File Generator. For each docking, the central atom and number of active bonds in ligand were detected and saved as. pdbqt file.

#### Molecular Docking

E6 structure and ligand molecules to be used for docking were defined, and the search space (Grid box) was specified using AutoGrid (part of the AutoDock package) around E6 molecule for a blind docking. A grid of 126, 126, and 126 points in the x, y, and z directions and the whole E6 molecule was centred by running the AutoGrid. Protein-ligand docking studies were performed using the AutoDock 4.2 program. The Genetic Algorithm was applied to model the interaction pattern between the E6 protein and the phytochemicals. For all docking procedures, 10 independent genetic algorithm runs with a population size of 150 were considered for each molecule under study. A maximum number of 2.5 × 10^5^ energy evaluations, 27,000 maximum generations, a gene mutation rate of 0.02, and a crossover rate of 0.8 were used. AutoDock was run in order to prepare corresponding docking log file (.dlg) for further analysis. The E6 as rigid molecule, ligand and docking parameters were defined and AutoDock4 was launched. The resultant log file was then used for further deriving the information pertaining to Binding Energies (BE) and Inhibition Constants (IC) of different conformations. The macromolecule and ligand complex was saved in. pdb format to be analyse in PyMOL.

#### Visualization

The visualization of structure files was done using the graphical interface of the ADT tool and the PyMol molecular graphics system and Discovery Studio Visualizer.

### Statistical Analysis

The data analysis was performed using the Microsoft Excel and IC50 was calculated using Prism (GraphPad). All cell culture experiments were carried out at least in 3 independent runs. Statistical significance of difference between the 2 test groups was analysed by the Student’s *t*-test and multiple comparisons versus control group was assessed by analysis of variance. In all cases, *p* value ≤ 0.05 was considered to be significant. The distribution of data set was normalized using Shapiro-Wilk normality test (*p* > 0.05). Further, we performed One way ANOVA and post hoc test for multiple comparisons.

## Results

### BAMT Induced a Dose-dependent Decline in CaCx Cell Viability

**FIGURE 1:**
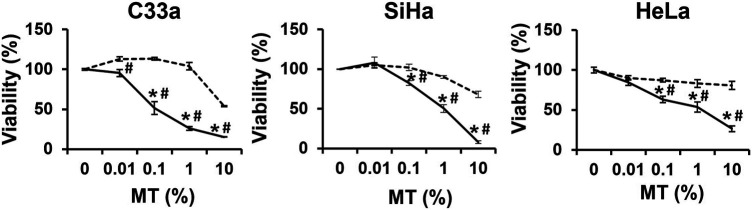
Effect of *Berberis aquifolium* mother tincture (BAMT) on the viability of different CaCx cell lines. Each panel shows percent cell viability of different HPV negative (C33a) and HPV positive (SiHa and HeLa) CaCx cell lines treated with increasing concentrations (0.01, 0.1, 1 and 10%) of MT of *B. aquifolium* at 24 h. The cell viability was measured by MTT assay as described in methods. Cells were similarly treated with succussed ethanol strength for MT- 70%) at corresponding concentration of alcohol content in the drug as alcohol/vehicle control. The results are representative of three independent experiments with similar results, data represent mean ± SD with *p values ≤ 0.05 with respect to control. ^#^ *p* values ≤ 0.05 compared to cultures treated with corresponding concentrations of vehicle control. Solid lines represent drug and broken lines represent vehicle control.

To assess the anti-cervical cancer response, cultures of CaCx cell lines, C33a, SiHa and HeLa were incubated with increasing dilutions of the drug (BAMT) for 24 h and the cell viability was evaluated by MTT assay. The BAMT-treated CaCx cell cultures, irrespective of their HPV status, showed a dose-dependent loss of cell viability >Figure 1). The cytotoxic response was also observable at higher dilutions (0.01 and 0.1%) in all the cell types and showed a distinct difference from the cytotoxicity displayed by the VC (IC50: C33a (BAMT–0.17% vs. VC–9.09%), SiHa (BAMT–0.65% vs. VC–14.12%) and HeLa (BAMT–0.67% vs. VC–17.28%)).

### BAMT Induces Cell Cycle Arrest at G0/G1 Checkpoint in CaCx Cells

**FIGURE 2:**
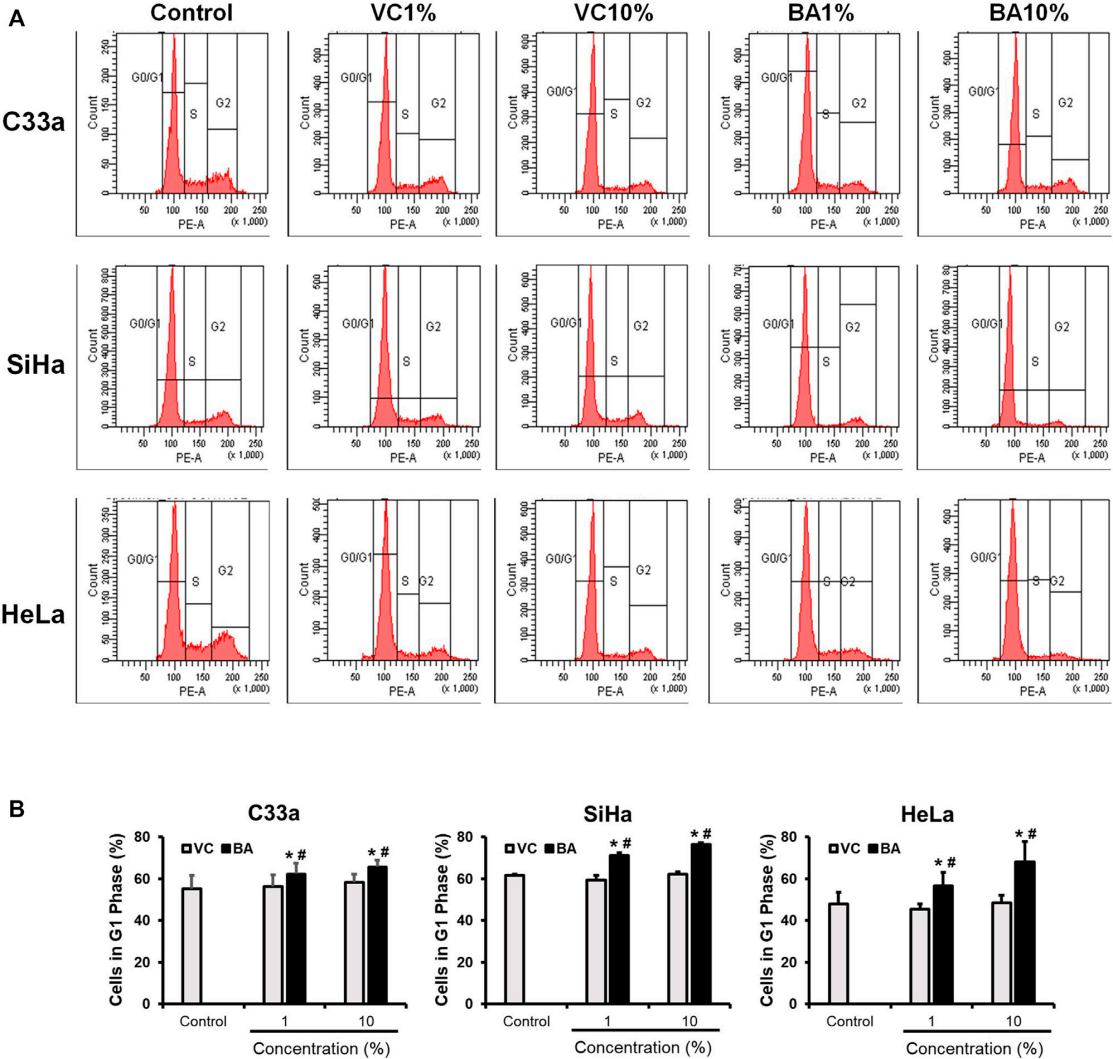
Effect of BAMT on distribution of cervical cancer cells in different phases of the cell cycle. **(A)** Representative flowcytometric analysis showing histograms of propidium iodide-stained nuclei from BAMT-treated CaCx cells. Cell were gated for G1, S and G2/M phases. **(B)**. Cumulative analysis of total cell population in G1 phase after treatment with BAMT or alcohol. Data represent mean ± SD with *p values ≤ 0.05 with respect to control. ^#^ *p* values ≤ 0.05 compared to cultures treated with corresponding vehicle control (VC).

Growth inhibitory effect of BAMT was investigated further to assess the distribution of cells in different phases of the cell cycle using flow cytometry. C33a SiHa and HeLa cells treated with BAMT (1 and 10%) or corresponding VC for 24 h showed a decline in the number of cells in S and G2/M phase and an equivalent increase in G0/G1 phase of the cell cycle (Figure 2A). Evaluation of changes in cell distribution in G1 phase in different experiments revealed a small but statistically significant increase in G0/G1 phase (Figure 2B). These differences were perceivable in live adherent cells at both the concentrations of BAMT tested (1 and 10%) as compared to cells treated with corresponding VC.

### BAMT Downregulated Expression of Oncogenic Transcription Factors in CaCx Cells

**FIGURE 3:**
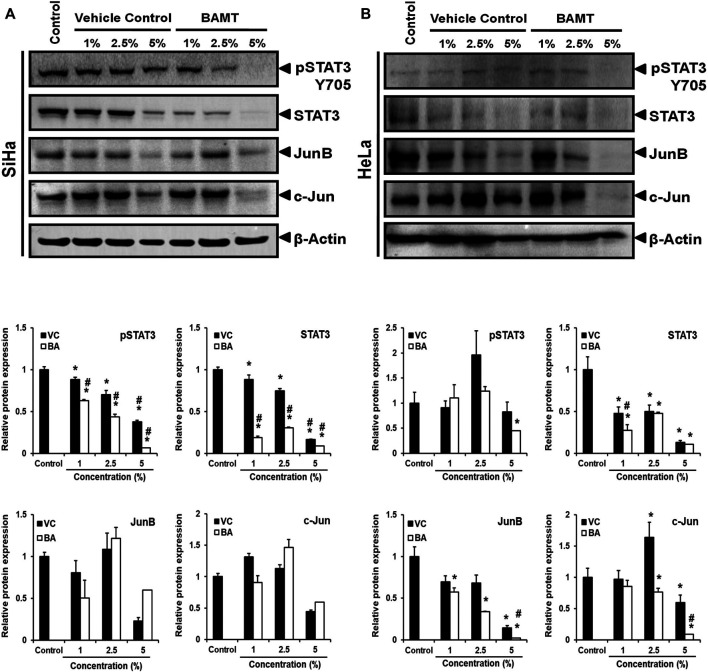
Effect of BAMT on expression of pSTAT3, STAT3 and AP-1 components, JunB and c-Jun. **(A)**. Representative immunoblots of total cellular proteins (40 µg/lane) isolated from BAMT-treated SiHa **(A)** or HeLa cells **(B)** separated on 10% SDS-PAGE were transferred on PVDF membrane and first probed for pSTAT3(Y705), and then reprobed for STAT3, JunB, c-Jun and β-actin **(upper panels)**. Graphs **(Lower panels)** show aggregated normalized fold change in band intensities of pSTAT3, STAT3, Jun B and c-Jun obtained from densitometric analysis. Values are means ± S.D. (indicated as error bars) of the three independent measurements. *p value <0.05 vs. control. ^#^ *p* value <0.05 vs. vehicle control (VC).

Next, we examined the level of transcription factors, STAT3 and AP-1 proteins, JunB and c-Jun in BAMT-treated CaCx cells. BAMT-treated cells irrespective of their infecting HPV genotype showed a dose-dependent decline in the level of pSTAT3(Y705) and STAT3 (Figure 3). However, it was intriguing to note that overall STAT3 level that included non-phosphorylated STAT3 declined sharply in contrast to pSTAT3. Though, cells treated with VC also showed a similar dose-response with reduced pSTAT3/STAT3 but the reduction was of a lower magnitude. The pSTAT3/STAT3 levels of treated HeLa cells were erratic and decline of pSTAT3/STAT3 levels were seen at BAMT (5%). BAMT-treated SiHa cells, on the other hand, failed to show significant changes in JunB and c-Jun level with respect to untreated or VC-treated cells and the response was inconsistent, whereas a specific decline in JunB and c-Jun was recorded for BAMT-treated HeLa cells.

### Effect of BAMT on Nuclear Localization of Oncogenic Transcription Factors in CaCx Cells

**FIGURE 4:**
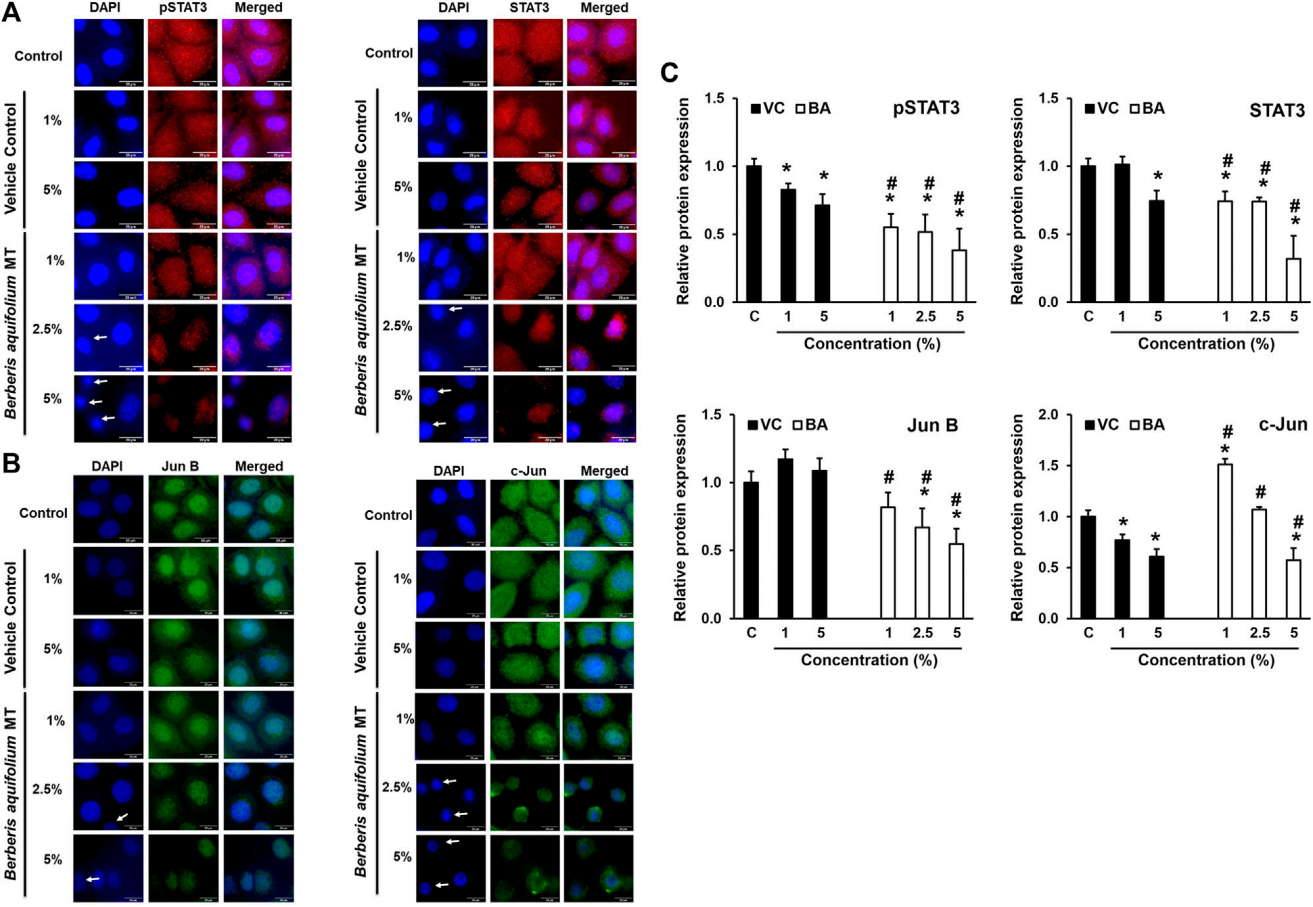
Localization of pSTAT3, STAT3, JunB and c-Jun in BAMT-treated SiHa cells. Representative fluorescence photomicrographs showing distribution of indicated transcription factors in BAMT-treated and control cells by immunocytochemistry. Cells were fixed, stained with respective antibodies and detected with goat anti-mouse IgG-Alexa-594 (for pSTAT3Y705 and STAT3; Red; **(A)** and goat anti-rabbit-IgG-Alexa-488 (Jun B and c-Jun; Green; **(B)** and counterstained with DAPI (Blue). Arrow indicates nuclei with reduced size. The expression level was measured by ImageJ and plotted as mean ± SEM of the representative experiment out of three independent experiments **(C)**. Values were obtained by analyzing at least 30 cells per specimen. *p value < 0.05 vs. control. ^#^ *p* value < 0.05 vs. vehicle control (VC).

BAMT-treated SiHa cells immune-stained for different transcription factors were subjected to fluorescence microscopy to visualize the localization of these transcription factors within the cell and their translocation into the nucleus (Figure 4). BAMT-treated SiHa cells showed dose-dependent decline in overall pSTAT3 and STAT3 staining. Both pSTAT3, STAT3 staining in cytoplasm significantly declined, whereas a relatively-reduced but still prominent staining was detected in the nuclei. A concomitant reduction in the nuclear size of many of the live cells in culture was noted in some microscopic fields. Strong positivity of JunB in the nuclei and a diffused positivity of c-Jun well distributed in cytoplasm and nuclei was detected in control cells. Following BAMT-treatment, a marked decline in JunB and c-Jun was noted (Figure 4C). However, there was no notable re-distribution of these AP-1 components was observed in BAMT-treated cells at any strength of drug tested (Figures 4A,B).

### BAMT Reduced the Expression of HPV Oncoproteins E6 and E7 in CaCx Cells

**FIGURE 5:**
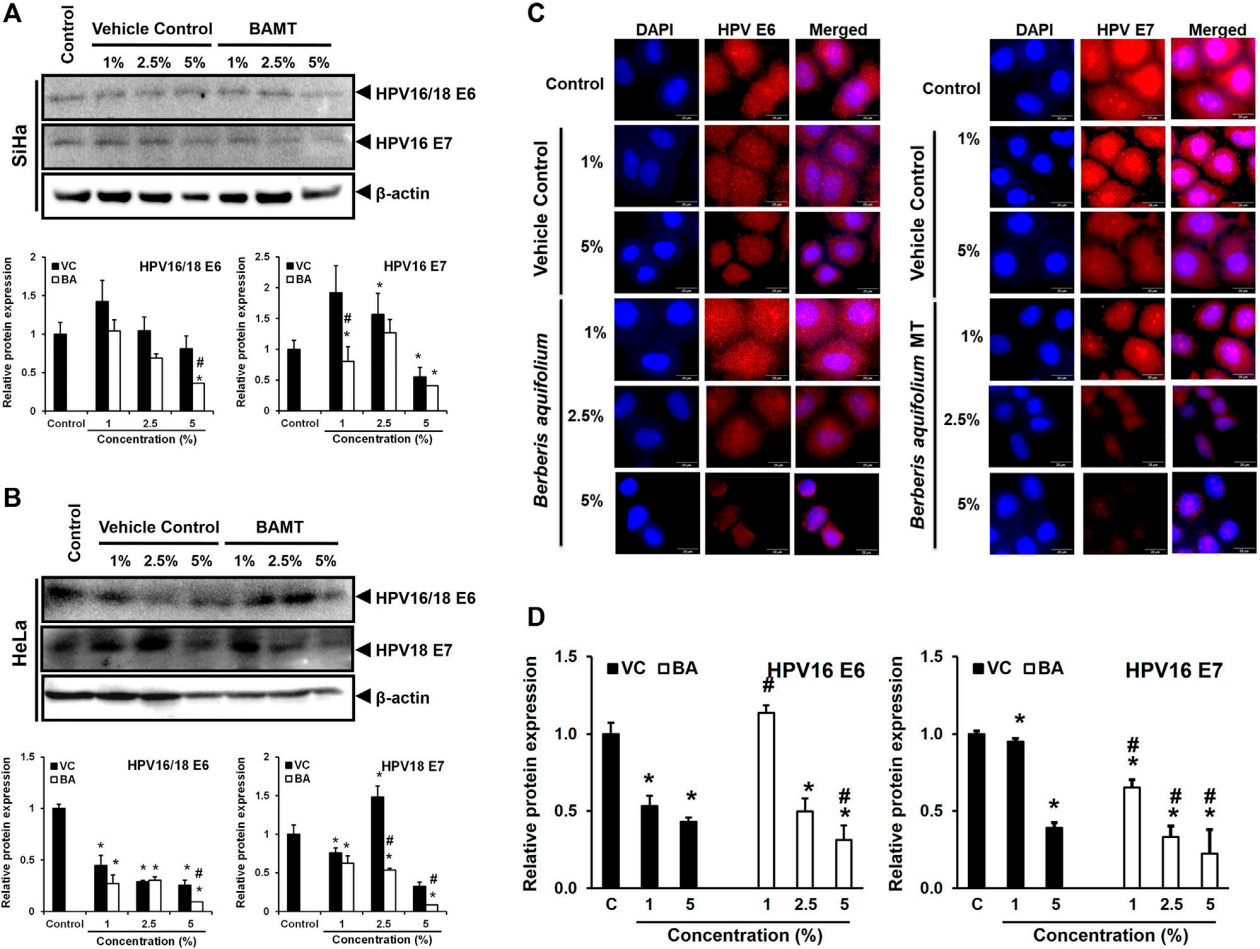
Effect of BAMT on expression of HPV oncoproteins E6 and E7 and localization. A. and B. Lower blot (with ≤25 kDa proteins) of the representative experiment shown in Figure 3. BAMT-treated SiHa **(A)** or HeLa cells **(B)** were first probed for HPV E6, and then re-probed for HPV E7 **(upper panels)**. *β-actin of corresponding upper blot is reproduced for comparison. Graphs **(Lower panels)** show aggregated normalized fold change in band intensities of HPV E6 and HPV E7 obtained from densitometric analysis. Values are means ± S.D. (indicated as error bars) of the three independent measurements. **C. and D.** Representative fluorescence photomicrographs showing distribution of HPV16 E6 and E7 in BAMT-treated and control SiHa cells by immunocytochemistry **(C)**. Cells were fixed, stained with HPV16/18 E6 and HPV16 E7 antibodies and detected with goat anti-mouse IgG-Alexa-594 (Red) and counterstained with DAPI (Blue). The expression level was measured by ImageJ and plotted as mean ± SEM of the representative experiment out of three independent experiments **(D)**. Values were obtained by analyzing at least 30 cells per specimen. *p value < 0.05 vs. control. ^#^ *p* value < 0.05 vs. vehicle control (VC).

To assess the downstream effect of BAMT on level of viral oncoproteins in treated cells, the lower part of the membranes derived from immunoblotting experiments with proteins ≤25 kDa were used to measure transcription factor expression were probed for HPV16/18 E6 and E7 (Figures 5A,B). A dose-dependent decline of both HPVE6 and E7 proteins in SiHa and HeLa cells was noted, but the response achieved statistical significance only at higher concentrations (2.5 and 5%). The inhibition of E6 and E7 expression was stronger for HeLa cells and was only marginally better than the VC in SiHa cells. Immunocytochemical staining of SiHa cells further confirmed the loss of E6 and E7 proteins (Figure 5C). However, the nuclear positivity of E6 and E7 was not affected in BAMT-treated cells.

### Anti-Oncogenic Potential of Phytochemicals Presents in BAMT on HPV E6 Activity

**FIGURE 6:**
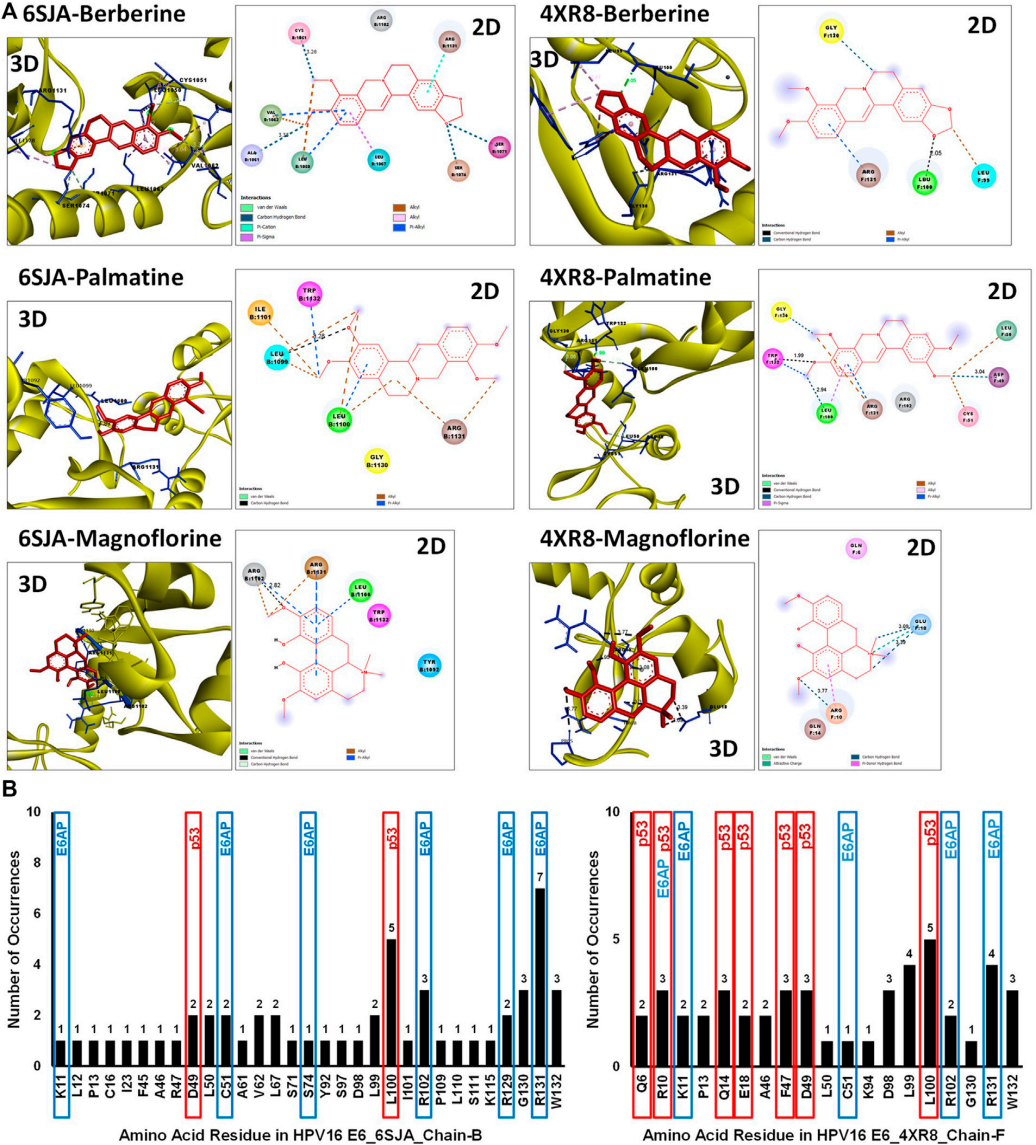
Molecular docking of key phytochemicals reported in *B. aquifolium* root with HPV16 E6 and its impact of their binding on E6’s carcinogenic interaction. **(A)**. Best ranked docking pose (3D) and a 2D simplified representation of the binding pockets of three best leads, berberine, palmatine and magnoflorine, in association with HPV16 E6 (based on 6SJA (**left panel**) or 4XR8 (**right panel**) crystal structure) showing interacting residues and non-covalent interactions. The hydrogen bonds are shown as dashed lines. **(B)**. Graphical representation of the cumulative data showing number of times an amino acid residue of HPV16 E6 (6SJA (**left panel**) or 4XR8 (**right panel**)) appeared in the binding pocket of different phytochemical ligands examined. The residues identified in molecular docking that are involved in key carcinogenic interactions with p53 or E6AP are highlighted with different colored boxes.

The disparity between strong inhibitory effect of BAMT and moderate effect on HPV16 E6 and E7 was investigated further using *in silico* molecular docking approach. Phytochemicals reportedly present in root of *B. aquifolium* root were downloaded and a blind molecular docking was performed against two most recent high-resolution crystal structures of HPV16 E6 present in PDB, 6SJA (chain B) and 4RX8 (chain F) Supplementary Figures SF2A, SF2B. Molecular docking characteristics of each phytochemical and the interacting amino acid residues with potential binding partners in the HPV16 E6 binding pocket were identified. The carcinogenically-relevant amino acid residues were determined using reported key interacting motifs and participating amino acid residue for p53, E6AP, PDZ, and Zn-binding domain (Supplementary Table ST2). Among the top 10 confirmations obtained for each phytochemical from docking experiments (Supplementary Tables ST3, ST4), only the best-ranked docking conformation with lowest binding energy are described in Table 1 Berberine showed a strong interaction with 6SJA but showed a moderate binding to 4XR8 structure. Notably, magnoflorine and palmatine showed a stronger specific interaction with E6 than berberine and manifested high negative binding energies and low inhibition constants (Figure 6A). Most of the interactions involved either p53 interacting pocket (columbamine), or E6AP interacting pocket of E6 (obamegine and aromoline), or both (berbamine, palmatine, jatrorrhizine, magnoflorine, oxyacanthine) with different affinities (Table 1). In case of E6 conformation 4XR8, most of the interactions involved amino acid residues that were common to p53 and E6AP binding pockets, though the corresponding negative binding energies were comparatively less than their respective interaction in E6 (6SJA) (Table 1). Cumulative analysis of all interacting amino acid residue revealed, L100 and R131 as the most targeted residues of E6 in both the structures that participated in interaction with p53 and E6AP, respectively (Figure 6B).

**TABLE 1:** Molecular docking characteristics of various phytochemicals reportedly present in root of *B. aquifolium* with the 3D crystallographic structure of HPV16 E6 (6SJA and 4XR8) available on PDB and their interacting amino acid residues with potential binding partners in the HPV16 E6 binding pocket.

S. No	Ligand name^a^	Activities reported	Nature	PubChem CID	6SJA (chain B) (best ranked docking conformation)				4XR8 (chain F) (best ranked docking conformation)			
					BE (kcal/mol)	IC (µM)	AA residues^c^	Known E6 interacting partner(s)	BE (kcal/mol)	IC (µM)	AA residues^c^	Known E6 interacting partner(s)
1	Berberine	130	Alkaloid	CID 2353	−6.6	13.7	L50, *C51*, A61, V62, L67, S71, *S74*, *R102*, *R131*	*E6AP*	-5.1	193.4	L99, **L100**, G130, *R131*	**p53*,*** *E6AP*
2	Berbamine	38	Alkaloid	CID 275182	−6.1	31.7	*K11*, **D49**, S97, S98, L99, **L100**, P109, L110, S111, *K11*5	**p53,** *E6AP*	-3.9	1,460	***R10***, D98, L99, **L100**, *R131*	**p53*,*** *E6AP*
3	Palmatine	17	Isoquiniline alkaloid	CID 19009	−6.7	12.6	L99, **L100**, I101, G130, *R131*, W132	**p53**,*E6AP*	-5.7	66.4	**F47**, **D49**, L50, *C51*, **L100**, *R102*, G130, *R131*, W132	**p53*,*** *E6AP*
4	Hydrastine	16	Alkaloid	CID 197835	−4.5	476.2	F45, L50, *C51*, V62, L67, *R102*	*E6AP*	-4.0	1,220	*K11*, P13, **Q14**, A46, **F47**, **D49**	**p53*,*** *E6AP*
5	Jatrorrhizine	13	Protoberberine alkaloid	CID 72323	−6.0	40.9	**L100**, *R131*, W132	**p53**, *E6AP*	-5.4	110.2	**Q6**, ***R10***, **Q14**, **E18**	**p53** *, E6AP*
6	Magnoflorine	11	Apophine alkaloid	CID 73337	−7.1	6.5	Y92, **L100**, *R102*, *R131*, W132	**p53**, *E6AP*	-5.6	80.9	**Q6**, ***R10***, **Q14**, **E18**	**p53** *, E6AP*
7	Oxycanthine	10	Alkaloid	CID 442333	−6.1	36.7	**L100**, G130, *R131*	**p53**, *E6AP*	-3.9	1,500	D98, L99, **L100**	**p53**
8	Columbamine	7	Berberine alkaloid	CID 72310	−6.4	21.8	L12, P13, C16, I23, A46, R47,**D49**	**p53**	-5.2	144.3	L99, **L100**, *R102*, *R131*, W132	**p53** *, E6AP*
9	Obamigine	4	Phenolic base	CID 441064	−5.6	83.9	*R129*, G130, *R131*	*E6AP*	-4.2	831.3	*K11*, P13, A46, **F47**, **D49**	**p53** *, E6AP*
10	Aromaline	2	Alkaloid	CID 362574	−6.2	30.5	*R129*, *R131*	*E6AP*	-4.1	1,020	K94, D98, L99, **L100**, *R131*, W132	**p53** *, E6AP*

a From Dr. Duke’s Phytochemical and Ethnobotanical database (USDA 1992–2016)

b High negative binding energy is indicative of non-specific interaction.

c Bold amino acid residues correspond to p53 binding pocket, italic amino acid residue corresponds to E6AP-binding pocket. BE: binding energy, IC: inhibitory constant.

## Discussion

In this report, we show the presence of anti-CaCx and anti-HPV activity in the *B. aquifolium*-based herbal preparation. BAMT induced a dose-dependent decline in the viable CaCx cells irrespective of their HPV status. Remnant BAMT-treated cells showed G1 phase growth arrest. BAMT induced a decline in STAT3, JunB and c-Jun, however, their nuclear localization was not affected. Reduction in STAT3/pSTAT3 level was more consistent in SiHa cells, whereas, alterations in AP1 members were consistent in HeLa cells suggesting different targets/mechanisms of inhibition. Nevertheless, a decline in HPV E6/E7 expression in CaCx cells irrespective of the cell type was noted in association with loss of the transcription factors in BAMT-treated cells. Using *in silico* molecular docking we show for the first time presence of anti-HPV activity in phytochemicals of BAMT that could strongly interfere with the normal function of HPV16 E6 to bind p53 and E6AP. We identified leading BAMT-constituent molecules showing highest negative free energy for cooperative binding to HPV16 E6.

The cell viability study demonstrated that anti-CaCx activity reported in berberine (Jantova et al., 2003; Mahata et al., 2011; Chu et al., 2014; Saha and Khuda-Bukhsh 2014), is well represented in BAMT. Early studies showed *B. aquifolium* constituents to be effective in preventing cell growth of human keratinocytes during psoriasis (Muller et al., 1995), a pro-inflammatory hyperproliferative disease. However, direct effect of BAMT on cervical cancer has not been reported. In a study where HeLa cells were treated with *B. aquifolium* ethanolic extracts that resembled BAMT, showed similar growth inhibition with comparable IC_50_ values (Damjanović et al., 2016). Interestingly, berberine specifically targeted HPV-positive CaCx cells (Mahata et al., 2011), whereas, BAMT could prevent the growth of both HPV-positive and HPV-negative CaCx cells. The reasons behind broader spectrum of BAMT’s response are not known. Besides berberine, ethanolic extracts of *B. aquifolium* root are known to contain high concentrations of other metabolites like berbamine, palmatine, hydrastine, jatrorrhizine, oxyacanthine, columbamine, obamegine, and aromoline (Godevac et al., 2018, USDA 1992–2016) that could possibly contribute to the anti-cancer effects of BAMT. BAMT has been reported to contain high alkaloid content equivalent to 5.5 mg/ml of berberine but a net content of berberine is only 0.5 mg/ml (Ungurean et al., 2018). Therefore, the resultant effect seen in BAMT-treated CaCx cells could be multifactorial and could be contributed by phytochemicals other than berberine. Indeed, many phytochemical constituents like berbamine (Wang et al., 2009), palmatine (Hagiwara et al., 2015; Liu et al., 2020), hydrastine (Guo et al., 2016), jatrorrhizine (Liu et al., 2013), magnoflorine (Sun et al., 2020; Wei et al., 2020), and columbamine (Bao et al., 2012) have displayed anti-cancer properties in different tumor models.

Induction of cell death is a major contributor to reduced cell growth in BAMT-treated cells as observed in the MTT assay. We were particularly interested in cell cycling state of BAMT-treated cells, which survived BAMT and remained attached in cultures. These cultures showed a small but significant increase in proportions of CaCx cells in G1-phase in all cell types tested. Berberine is known to induce cell cycle arrest predominantly in G1-phase (Eom et al., 2008; Yan et al., 2011; Puthdeei et al., 2017; Gu et al., 2020). However, G2/M arrest in berberine-treated cancer cells is also reported (Lin et al., 2006; Mantena et al., 2006; El Khalki et al., 2020) and the phenomenon was found to vary with berberine’s dose and the cell type. Interestingly, when berberine is used to treat Hela cells, G1 growth arrest was noted (Gu et al., 2020). Apart from berberine, berbamine (Zhang et al., 2018), jatrorrhizine (Liu et al., 2013) and hydrastine (Guo et al., 2016) are reported to induce G1 arrest, whereas, columbamine (Bao et al., 2012), palmatine (Liu et al., 2020) and magnoflorine (Sun et al., 2020) induced cell growth arrest in G2/M phase in different *in vitro* cell culture models. Despite variations in mechanisms of cell cycle arrest, our study shows that BAMT-induces G1 growth arrest that could be the resultant effect of multifactorial inhibition of cellular growth on different checkpoints, which could be less toxic as predicted earlier for other phytochemicals (Singh et al., 2002).

Our data revealed for the first time that BAMT-induced alterations in expression of STAT3 and AP-1 that control expression of viral oncogenes E6 and E7 and thereby contributing to BAMT’s anti-CaCx activity. Notably, STAT3 inhibition was prominent in SiHa, whereas AP-1 inhibition prevailed in HeLa cells. BAMT-could not block the upstream signalling that would have resulted in their cytoplasmic retention. This shows that BAMT exerted its inhibitory effects by different mechanisms in different cell lines. Though, the mechanisms by which BAMT led to loss of STAT3/pSTAT3 or JunB/c-Jun, are currently not known, but can be attributed to the constituent phytochemicals. We earlier showed that berberine inhibits AP-1 and STAT3 (Mahata et al., 2011; Pandey et al., 2015), however, in those studies berberine targeted the activation state of these transcription factors. A number of studies showed a decline in pSTAT3(Y705) levels and STAT3 activity that were stronger than the effect on total cellular levels of STAT3 (Pandey et al., 2015; Zhu et al., 2015; Puthdee et al., 2017). These studies showed moderate effect of total STAT3 pools in the berberine-treated cells. Similar targeting of STAT3 signaling was reported with berbamine (Hu et al., 2019), and columbamine (Bao et al., 2012). The reasons underlying enhanced degradation of unphosphorylated STAT3 compared to the pSTAT3 pool that usually get translocated to the nucleus in BAMT-treated cells is not known presently. However, a calcineurin-mediated enhanced proteasomal degradation of cytoplasmic STAT3 is expected as reported earlier (Murase 2013). Berbamine was shown to increase intracellular calcium, which activates calcineurin.

Contrary to the BAMT’s effect on STAT3, two key components of AP-1 signaling, JunB and c-Jun were severely affected in BAMT-treated cells. Earlier we showed berberine inhibited only c-Jun level particularly in HeLa cells (Mahatai et al., 2011). Here, BAMT also downregulated c-Jun in HeLa cells. However, in BAMT-treated SiHa cells the alteration in c-Jun was not significant. The composition of AP-1 complex varies with cell lines (Mahata et al., 2011), Under normal conditions, SiHa cell line shows AP-1 complex with c-Fos, JunB, and JunD, while in HeLa cells c-Jun participates actively in the AP1 complex making the underlying variation a cause of differential response. In contrast to berberine, magnoflorine was shown to induce AP-1 signalling by enhancing JNK activity in a different cell type (Sun et al., 2020). Nevertheless, BAMT induced loss of expression of key components of transcriptional machinery that translated into their reduced availability to promote oncogenic transcription. Further, we report here a decline in the expression of viral oncoproteins E6 and E7 following BAMT treatment. These observations are similar to our previous study on berberine (Mahata et al., 2011). However, the extent of inhibition was relatively lesser and did not match to cytotoxic action of BAMT. Moreover, nuclear localization of the oncogenes was noted despite the loss of their gene expression. Nuclear localization of HPV16 E6 due to 3 nuclear localization signals present on this protein has been reported earlier (Tao et al., 2003). This suggests that there could be alternate mechanisms other than targeting expression of the viral oncogenes that are exerted by BAMT in inducing growth arrest and cell death in CaCx.

To address the disparity, in the next part of the investigation we examined potential interaction of BAMT constituents with HPV16 E6 by molecular docking experiments to evaluate their cooperative binding. Blind docking of these phytochemicals on two latest HPV16 E6 crystal structures available on PDB (6SJA and 4XR8) revealed a strong interaction of berberine, magnoflorine and palmatine to E6 and the binding pocket overlapped aa residues reportedly involved in E6’s interaction with p53 and E6AP (Zanier et al., 2013; Martinez-Zapien et al., 2016). The results were consistent irrespective of inherent structural differences due to their co-crystallized chains, where 4XR8 showed a conformation with slightly lower resolution (2.55 Å vs. 1.50 Å) compared to 6SJA, was co-crystallized with p53 (Martinez-Zapien et al., 2016). On the other hand, 6SJA co-crystallized with IRF3 LxxLL motif and possibly represented p53 unbound form (Suarez et al. 2019). Global alignment of the two E6 sequences show 98% identity with 2 gaps and replacement of arginine at 49th position of 6SJA with phenylalanine at 47th position of 4XR8. Both the structures had mutations 4 cysteine residues that interacted with Zn. For the present investigation, we specifically used GA algorithm in AutoDoc4. GA algorithm is helps differentiate between false positives and true negatives from the clusters as it gives more pose (conformers) and hence better than LGA, which is frequently used recently by some of the investigators (Kumar et al., 2015; Kolluru et al., 2019; Nabati et al., 2020). Although the leads from docking experiments are extremely valuable to support BAMT’s potential action on E6’s physiological activity, detailed wet-lab experiments are essentially needed to validate these results in the experimental setup. Though it is tempting to speculate that similar inhibitions may also be observed in other HR or LR-HPV E6 due to structural similarity, the current finding cannot be used to extrapolate/predict the same for HR/LR-HPV types.

## Summary and Conclusion

**FIGURE 7:**
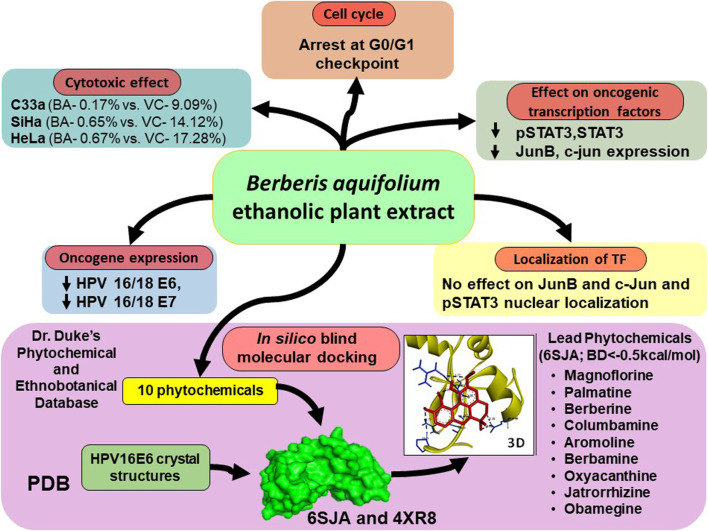
Schematic representation of multifactorial action of BAMT on CaCx cells.

Phytochemicals and herbal preparations are globally recognized as the second line of therapy after allopathy. These medicines are economic and there are no known side effects of the therapy. Taking these aspects into consideration, present study demonstrates a multifactorial action of BAMTon CaCx cells. BAMT induced cell death and G1 growth arrest in CaCx cells irrespective of their HPV status. Molecularly, these phytochemicals targeted oncogenically-relevant transcription factors of STAT3 and AP-1 family that resulted in loss of oncoprotein expression. Among various constituents, berberine, magnoflorine and palmatine were found capable of targeting E6 functions by strong cooperative binding Figure 7. Collectively, these leads show that commercially-available BAMT can be an effective and economic broad-spectrum therapeutic option against cervical cancer.

## Data Availability

The original contributions presented in the study are included in the article/Supplementary Material, further inquiries can be directed to the corresponding author/s.

## Abbreviations

BA, *Berberis aquifolium*; CaCx, cervical cancer; HPV, human papillomavirus; MT, mother tincture; OD, optical density; PI, propidium iodide; VC, vehicle control.

## References

[B1] Angel P. Imagawa M. Chiu R. Stein B. Imbra R. J. Rahmsdorf H. J. (1987). Phorbol Ester-Inducible Genes Contain a Common Cis Element Recognized by a TPA-Modulated Trans-acting Factor. Cell 49 (6), 729–739. 10.1016/0092-8674(87)90611-8 3034432

[B2] Arbyn M. Weiderpass E. Bruni L. de Sanjosé S. Saraiya M. Ferlay J. (2020). Estimates of Incidence and Mortality of Cervical Cancer in 2018: a Worldwide Analysis. Lancet Glob. Health 8 (2), e191–e203. 10.1016/S2214-109X(19)30482-6 31812369 PMC7025157

[B3] Bao M. Cao Z. Yu D. Fu S. Zhang G. Yang P. (2012). Columbamine Suppresses the Proliferation and Neovascularization of Metastatic Osteosarcoma U2OS Cells with Low Cytotoxicity. Toxicol. Lett. 215 (3), 174–180. 10.1016/j.toxlet.2012.10.015 23124089

[B4] Bharti A. C. Donato N. Singh S. Aggarwal B. B. (2003). Curcumin (Diferuloylmethane) Down-Regulates the Constitutive Activation of Nuclear Factor-Kappa B and IkappaBalpha Kinase in Human Multiple Myeloma Cells, Leading to Suppression of Proliferation and Induction of Apoptosis. Blood 101 (3), 1053–1062. 10.1182/blood-2002-05-1320 12393461

[B5] Bharti A. C. Singh T. Bhat A. Pande D. Jadli M. (2018). Therapeutic Startegies for Human Papillomavirus Infection and Associated Cancers. Front. Biosci. (Elite Ed. 10, 15–73. 10.2741/e808 28930604

[B6] Boericke W. (2001). New Manual of Homoeopathic Materia Medica with Repertory. New Delhi, B: Jain Publishers.

[B7] Bown D. Herb Society of America (1995). Encyclopedia of Herbs & Their Uses. London; New York; Boston: Dorling Kindersley; Distributed by Houghton Mifflin.

[B8] Bromberg J. F. Wrzeszczynska M. H. Devgan G. Zhao Y. Pestell R. G. Albanese C. (1999). Stat3 as an Oncogene. Cell 98 (3), 295–303. 10.1016/s0092-8674(00)81959-5 10458605

[B9] Chevallier A. (1996). The Encyclopedia of Medicinal Plants. New York, Boston: DK PubDistributed by Houghton Mifflin.

[B10] Chu S. C. Yu C. C. Hsu L. S. Chen K. S. Su M. Y. Chen P. N. (2014). Berberine Reverses Epithelial-To-Mesenchymal Transition and Inhibits Metastasis and Tumor-Induced Angiogenesis in Human Cervical Cancer Cells. Mol. Pharmacol. 86 (6), 609–623. 10.1124/mol.114.094037 25217495

[B11] Damjanović A. Zdunić G. Šavikin K. Mandić B. Jadranin M. Matić I. Z. (2016). Evaluation of the Anti-cancer Potential of Mahonia Aquifolium Extracts via Apoptosis and Anti-angiogenesis. Bangladesh J. Pharmacol. 11, 741–749.

[B12] Duke J. A. Ayensu E. S. (1985). Medicinal Plants of China. Algonac, Mich. Algonac, Michigan: Reference Publications.

[B13] El Khalki L. Maire V. Dubois T. Zyad A. (2020). Berberine Impairs the Survival of Triple Negative Breast Cancer Cells: Cellular and Molecular Analyses. Molecules 25 (3). 10.3390/molecules25030506 PMC7036777 31991634

[B14] Eom K. S. Hong J. M. Youn M. J. So H. S. Park R. Kim J. M. (2008). Berberine Induces G1 Arrest and Apoptosis in Human Glioblastoma T98G Cells through Mitochondrial/caspases Pathway. Biol. Pharm. Bull. 31 (4), 558–562. 10.1248/bpb.31.558 18379040

[B15] Godevac D. Damjanovic A. Stanojkovic T. P. Andelkovic B. Zdunic G. (2018). Identification of Cytotoxic Metabolites from Mahonia Aquifolium Using (1)H NMR-Based Metabolomics Approach. J. Pharm. Biomed. Anal. 150, 9–14. 10.1016/j.jpba.2017.11.075 29202306

[B16] Gu S. Song X. Xie R. Ouyang C. Xie L. Li Q. (2020). Berberine Inhibits Cancer Cells Growth by Suppressing Fatty Acid Synthesis and Biogenesis of Extracellular Vesicles. Life Sci. 257, 118122. 10.1016/j.lfs.2020.118122 32702446

[B17] Guo B. Li X. Song S. Chen M. Cheng M. Zhao D. (2016). (-)-β-hydrastine Suppresses the Proliferation and Invasion of Human Lung Adenocarcinoma Cells by Inhibiting PAK4 Kinase Activity. Oncol. Rep. 35 (4), 2246–2256. 10.3892/or.2016.4594 26821251

[B18] Hagiwara K. Gailhouste L. Yasukawa K. Kosaka N. Ochiya T. (2015). A Robust Screening Method for Dietary Agents that Activate Tumour-Suppressor microRNAs. Sci. Rep. 5, 14697. 10.1038/srep14697 26423775 PMC4589759

[B19] Hu B. Cai H. Yang S. Tu J. Huang X. Chen G. (2019). Berbamine Enhances the Efficacy of Gefitinib by Suppressing STAT3 Signaling in Pancreatic Cancer Cells. Onco Targets Ther. 12, 11437–11451. 10.2147/OTT.S223242 31920333 PMC6935307

[B20] Huibregtse J. M. Scheffner M. Howley P. M. (1991). A Cellular Protein Mediates Association of P53 with the E6 Oncoprotein of Human Papillomavirus Types 16 or 18. EMBO J. 10 (13), 4129–4135. 10.1002/j.1460-2075.1991.tb04990.x 1661671 PMC453163

[B21] IARC, W.I. W. Group (2012). Human Papillomaviruses. Biological Agents: A Review of Human Carcinogenes, 100B. Lyon, France: IACR, 255–313.

[B22] Jantová S. Cipák L. Kost'álová M. D. Kost'alova D. (2003). Effect of Berberine on Proliferation, Cell Cycle and Apoptosis in HeLa and L1210 Cells. J. Pharm. Pharmacol. 55 (8), 1143–1149. 10.1211/002235703322277186 12956905

[B23] Kolluru S. Momoh R. Lin L. Mallareddy J. R. Krstenansky J. L. (2019). Identification of Potential Binding Pocket on Viral Oncoprotein HPV16 E6: a Promising Anti-cancer Target for Small Molecule Drug Discovery. BMC Mol. Cel Biol 20 (1), 30. 10.1186/s12860-019-0214-3 PMC6685234 31387520

[B24] Kumar S. Jena L. Sahoo M. Kakde M. Daf S. Varma A. K. (2015). In Silico Docking to Explicate Interface between Plant-Originated Inhibitors and E6 Oncogenic Protein of Highly Threatening Human Papillomavirus 18. Genomics Inform. 13 (2), 60–67. 10.5808/GI.2015.13.2.60 26175664 PMC4500800

[B25] Lin C. C. Lin S. Y. Chung J. G. Lin J. P. Chen G. W. Kao S. T. (2006). Down-regulation of Cyclin B1 and Up-Regulation of Wee1 by Berberine Promotes Entry of Leukemia Cells into the G2/M-phase of the Cell Cycle. Anticancer Res. 26 (2A), 1097–1104. 10.1096/fasebj.20.5.a1131-c 16619512

[B26] Liu R. Cao Z. Pan Y. Zhang G. Yang P. Guo P. (2013). Jatrorrhizine Hydrochloride Inhibits the Proliferation and Neovascularization of C8161 Metastatic Melanoma Cells. Anticancer Drugs 24 (7), 667–676. 10.1097/CAD.0b013e328361ab28 23695011

[B27] Liu X. Zhang Y. Wu S. Xu M. Shen Y. Yu M. (2020). Palmatine Induces G2/M Phase Arrest and Mitochondrial-Associated Pathway Apoptosis in colon Cancer Cells by Targeting AURKA. Biochem. Pharmacol. 175, 113933. 10.1016/j.bcp.2020.113933 32224138

[B28] Mahata S. Bharti A. C. Shukla S. Tyagi A. Husain S. A. Das B. C. (2011). Berberine Modulates AP-1 Activity to Suppress HPV Transcription and Downstream Signaling to Induce Growth Arrest and Apoptosis in Cervical Cancer Cells. Mol. Cancer 10, 39. 10.1186/1476-4598-10-39 21496227 PMC3098825

[B29] Mantena S. K. Sharma S. D. Katiyar S. K. (2006). Berberine, a Natural Product, Induces G1-phase Cell Cycle Arrest and Caspase-3-dependent Apoptosis in Human Prostate Carcinoma Cells. Mol. Cancer Ther. 5 (2), 296–308. 10.1158/1535-7163.MCT-05-0448 16505103

[B30] Martinez-Zapien D. Ruiz F. X. Poirson J. Mitschler A. Ramirez J. Forster A. (2016). Structure of the E6/E6AP/p53 Complex Required for HPV-Mediated Degradation of P53. Nature 529 (7587), 541–545. 10.1038/nature16481 26789255 PMC4853763

[B31] Moerman D. E. (1998). Native American Ethnobotany. Portland, or. Dordrecht, Netherlands: Timber Press.

[B32] Müller K. Ziereis K. Gawlik I. (1995). The Antipsoriatic Mahonia Aquifolium and its Active Constituents; II. Antiproliferative Activity against Cell Growth of Human Keratinocytes. Planta Med. 61 (1), 74–75. 10.1055/s-2006-958005 7700998

[B33] Murase S. (2013). Signal Transducer and Activator of Transcription 3 (STAT3) Degradation by Proteasome Controls a Developmental Switch in Neurotrophin Dependence. J. Biol. Chem. 288 (28), 20151–20161. 10.1074/jbc.M113.470583 23733189 PMC3711283

[B34] Nabati F. Moradi M. Mohabatkar H. (2020). In Silico analyzing the Molecular Interactions of Plant-Derived Inhibitors against E6AP, P53, and C-Myc Binding Sites of HPV Type 16 E6 Oncoprotein. Mol. Biol. Res. Commun. 9 (2), 71–82. 10.22099/mbrc.2020.36522.1483 32802901 PMC7382397

[B35] Pandey A. Vishnoi K. Mahata S. Tripathi S. C. Misra S. P. Misra V. (2015). Berberine and Curcumin Target Survivin and STAT3 in Gastric Cancer Cells and Synergize Actions of Standard Chemotherapeutic 5-Fluorouracil. Nutr. Cancer 67 (8), 1293–1304. 10.1080/01635581.2015.1085581 26492225

[B36] Prusty B. K. Das B. C. (2005). Constitutive Activation of Transcription Factor AP-1 in Cervical Cancer and Suppression of Human Papillomavirus (HPV) Transcription and AP-1 Activity in HeLa Cells by Curcumin. Int. J. Cancer 113 (6), 951–960. 10.1002/ijc.20668 15514944

[B37] Puthdee N. Seubwai W. Vaeteewoottacharn K. Boonmars T. Cha'on U. Phoomak C. (2017). Berberine Induces Cell Cycle Arrest in Cholangiocarcinoma Cell Lines via Inhibition of NF-Κb and STAT3 Pathways. Biol. Pharm. Bull. 40 (6), 751–757. 10.1248/bpb.b16-00428 28566619

[B38] Rösl F. Das B. C. Lengert M. Geletneky K. zur Hausen H. (1997). Antioxidant-induced Changes of the AP-1 Transcription Complex Are Paralleled by a Selective Suppression of Human Papillomavirus Transcription. J. Virol. 71 (1), 362–370. 10.1128/JVI.71.1.362-370.1997 8985358 PMC191059

[B39] Saha S. K. Khuda-Bukhsh A. R. (2014). Berberine Alters Epigenetic Modifications, Disrupts Microtubule Network, and Modulates HPV-18 E6-E7 Oncoproteins by Targeting P53 in Cervical Cancer Cell HeLa: a Mechanistic Study Including Molecular Docking. Eur. J. Pharmacol. 744, 132–146. 10.1016/j.ejphar.2014.09.048 25448308

[B40] Satyavathi G. V. Gupta A. K. Tandon N. (1987). Medicinal Plants of India. New Delhi, Indian Council of Medical Research.

[B41] Scheffner M. Werness B. A. Huibregtse J. M. Levine A. J. Howley P. M. (1990). The E6 Oncoprotein Encoded by Human Papillomavirus Types 16 and 18 Promotes the Degradation of P53. Cell 63 (6), 1129–1136. 10.1016/0092-8674(90)90409-8 2175676

[B42] Shishodia G. Verma G. Das B. C. Bharti A. C. (2018). miRNA as Viral Transcription Tuners in HPV-Mediated Cervical Carcinogenesis. Front. Biosci. Schol Ed. 10, 21–47. 10.2741/s499 28930517

[B43] Shukla S. Mahata S. Shishodia G. Pandey A. Tyagi A. Vishnoi K. (2013). Functional Regulatory Role of STAT3 in HPV16-Mediated Cervical Carcinogenesis. PLoS One 8 (7), e67849. 10.1371/journal.pone.0067849 23874455 PMC3715508

[B44] Shukla S. Shishodia G. Mahata S. Hedau S. Pandey A. Bhambhani S. (2010). Aberrant Expression and Constitutive Activation of STAT3 in Cervical Carcinogenesis: Implications in High-Risk Human Papillomavirus Infection. Mol. Cancer 9, 282. 10.1186/1476-4598-9-282 20977777 PMC2984472

[B45] Singh R. P. Dhanalakshmi S. Agarwal R. (2002). Phytochemicals as Cell Cycle Modulators-Aa Less Toxic Approach in Halting Human Cancers. Cell Cycle 1 (3), 156–161. 10.4161/cc.1.3.117 12429925

[B46] Suarez I. P. Cousido-Siah A. Bonhoure A. Mitschler A. Podjarny A. Trave G. (2019). Structure of HPV16 E6 Oncoprotein in Complex with IRF3 LxxLL Motif. Paris, France: Protein Data Bank. ResearchCollaboratoryforStructuralBioinformatics(RCSB). 10.2210/pdb6sja/pdb

[B47] Sun X. L. Zhang X. W. Zhai H. J. Zhang D. Ma S. Y. (2020). Magnoflorine Inhibits Human Gastric Cancer Progression by Inducing Autophagy, Apoptosis and Cell Cycle Arrest by JNK Activation Regulated by ROS. Biomed. Pharmacother. 125, 109118. 10.1016/j.biopha.2019.109118 32106366

[B48] Tao M. Kruhlak M. Xia S. Androphy E. Zheng Z. M. (2003). Signals that Dictate Nuclear Localization of Human Papillomavirus Type 16 Oncoprotein E6 in Living Cells. J. Virol. 77 (24), 13232–13247. 10.1128/jvi.77.24.13232-13247.2003 14645580 PMC296047

[B49] Ungurean C. Carpa R. Câmpean R. Maior M. C. Olah N.-K. (2018). Phytochemical and Microbial Analyses of Berberis Sp. Extracts. Rom. Biotech. Lett. 25 (6), 2132–2139. 10.25083/rbl/25.6/2132.2139

[B50] USDA (1992-2016). Dr. Duke's Phytochemical and Ethnobotanical Databases. Washington, DC: US Department of Agriculture, Agricultural Research Service.

[B51] Wang S. Liu Q. Zhang Y. Liu K. Yu P. Liu K. (2009). Suppression of Growth, Migration and Invasion of Highly-Metastatic Human Breast Cancer Cells by Berbamine and its Molecular Mechanisms of Action. Mol. Cancer 8, 81. 10.1186/1476-4598-8-81 19796390 PMC2765940

[B52] Wei T. Xiaojun X. Peilong C. (2020). Magnoflorine Improves Sensitivity to Doxorubicin (DOX) of Breast Cancer Cells via Inducing Apoptosis and Autophagy through AKT/mTOR and P38 Signaling Pathways. Biomed. Pharmacother. 121, 109139. 10.1016/j.biopha.2019.109139 31707337

[B53] Yan K. Zhang C. Feng J. Hou L. Yan L. Zhou Z. (2011). Induction of G1 Cell Cycle Arrest and Apoptosis by Berberine in Bladder Cancer Cells. Eur. J. Pharmacol. 661 (1-3), 1–7. 10.1016/j.ejphar.2011.04.021 21545798

[B54] Yeo-Teh N. S. L. Ito Y. Jha S. (2018). High-Risk Human Papillomaviral Oncogenes E6 and E7 Target Key Cellular Pathways to Achieve Oncogenesis. Int. J. Mol. Sci. 19 (6), 1706. 10.3390/ijms19061706 PMC6032416 29890655

[B55] Zanier K. Charbonnier S. Sidi A. O. McEwen A. G. Ferrario M. G. Poussin-Courmontagne P. (2013). Structural Basis for Hijacking of Cellular LxxLL Motifs by Papillomavirus E6 Oncoproteins. Science 339 (6120), 694–698. 10.1126/science.1229934 23393263 PMC3899395

[B56] Zhang H. Jiao Y. Shi C. Song X. Chang Y. Ren Y. (2018). Berbamine Suppresses Cell Viability and Induces Apoptosis in Colorectal Cancer via Activating P53-dependent Apoptotic Signaling Pathway. Cytotechnology 70 (1), 321–329. 10.1007/s10616-017-0146-8 28965196 PMC5809661

[B57] Zhu T. Li L. L. Xiao G. F. Luo Q. Z. Liu Q. Z. Yao K. T. (2015). Berberine Increases Doxorubicin Sensitivity by Suppressing STAT3 in Lung Cancer. Am. J. Chin. Med. 43 (7), 1487–1502. 10.1142/S0192415X15500846 26503561

