# Supplementary material for: Targeting Aberrant Expression of STAT3 and AP-1 Oncogenic Transcription Factors and HPV Oncoproteins in Cervical Cancer by Berberis aquifolium
Source: Front Pharmacol. 2021 Oct 28;12:757414. doi: 10.3389/fphar.2021.757414 (PMC8580881; doi:10.3389/fphar.2021.757414)
Supplement: Supplementary file 1 [file Table1.DOCX]

**Supplementary Table 1: List of antibodies used for immunoblotting and immunocytochemistry experiments.**

| **S. No.** | **Antibodies**  **(Mol. Wt.)** | **Source and Cat. No.** | **Origin and type** | **Dilution in immunoblotting** | **Dilution in immuno-cytochemistry** |
| --- | --- | --- | --- | --- | --- |
|  | pSTAT3 (Y705) (92 kDa) | BD Biosciences  (612357) | Mouse monoclonal IgG 2a, κ | 1:5000 | 1:1000 |
|  | STAT3  92 kDa) | BD Biosciences (610190) | Mouse monoclonal IgG_1_ | 1:5000 | 1:1000 |
|  | JunB  (39 kDa) | Santa Cruz  (sc-73) | Rabbit polyclonal IgG | 1:5000 | 1:1000 |
|  | c-Jun  (39 kDa) | Santa Cruz  (sc-45) | Rabbit polyclonal IgG | 1:5000 | 1:1000 |
|  | HPV 16/18 E6 (16/17 kDa) | Santa Cruz  (sc-460) | Mouse monoclonal IgG κ light chain | 1:2500 | 1:1000 |
|  | HPV 16 E7  (21 kDa) | Santa Cruz  (sc-264) | Mouse monoclonal IgG 2a, κ light chain | 1:2500 | 1:1000 |
|  | HPV 18 E7  (15 kDa) | Santa Cruz  (sc-365035) | Mouse monoclonal IgG_1_ κ light chain | 1:2500 | 1:1000 |
|  | β-actin  (42 kDa) | Sigma  (A1978) | Mouse monoclonal IgG_1_ | 1:5000 | - |
|  | Anti-mouse-HRP | Santa Cruz  (sc-2031) | Goat anti-mouse IgG | 1:5000 | - |
|  | Anti- Rabbit-HRP | Santa Cruz  (sc-2030) | Goat anti-rabbit IgG | 1:5000 | - |
|  | Anti-mouse-AlexaFluor 594 | Invitrogen  (A11005) | Goat polyclonal IgG | - | 1:1000 |
|  | Anti-rabbit-AlexaFluor 488 | Invitrogen  (A11008) | Goat polyclonal IgG | - | 1:1000 |

Protein marker: Precision Plus Protein Dual Color Standards (Bio-Rad) (Catalog# 161-0374)
