# Supplementary material for: Targeting Aberrant Expression of STAT3 and AP-1 Oncogenic Transcription Factors and HPV Oncoproteins in Cervical Cancer by Berberis aquifolium
Source: Front Pharmacol. 2021 Oct 28;12:757414. doi: 10.3389/fphar.2021.757414 (PMC8580881; doi:10.3389/fphar.2021.757414)
Supplement: Supplementary file 2 [file Presentation1.PPTX]

## Slide 1
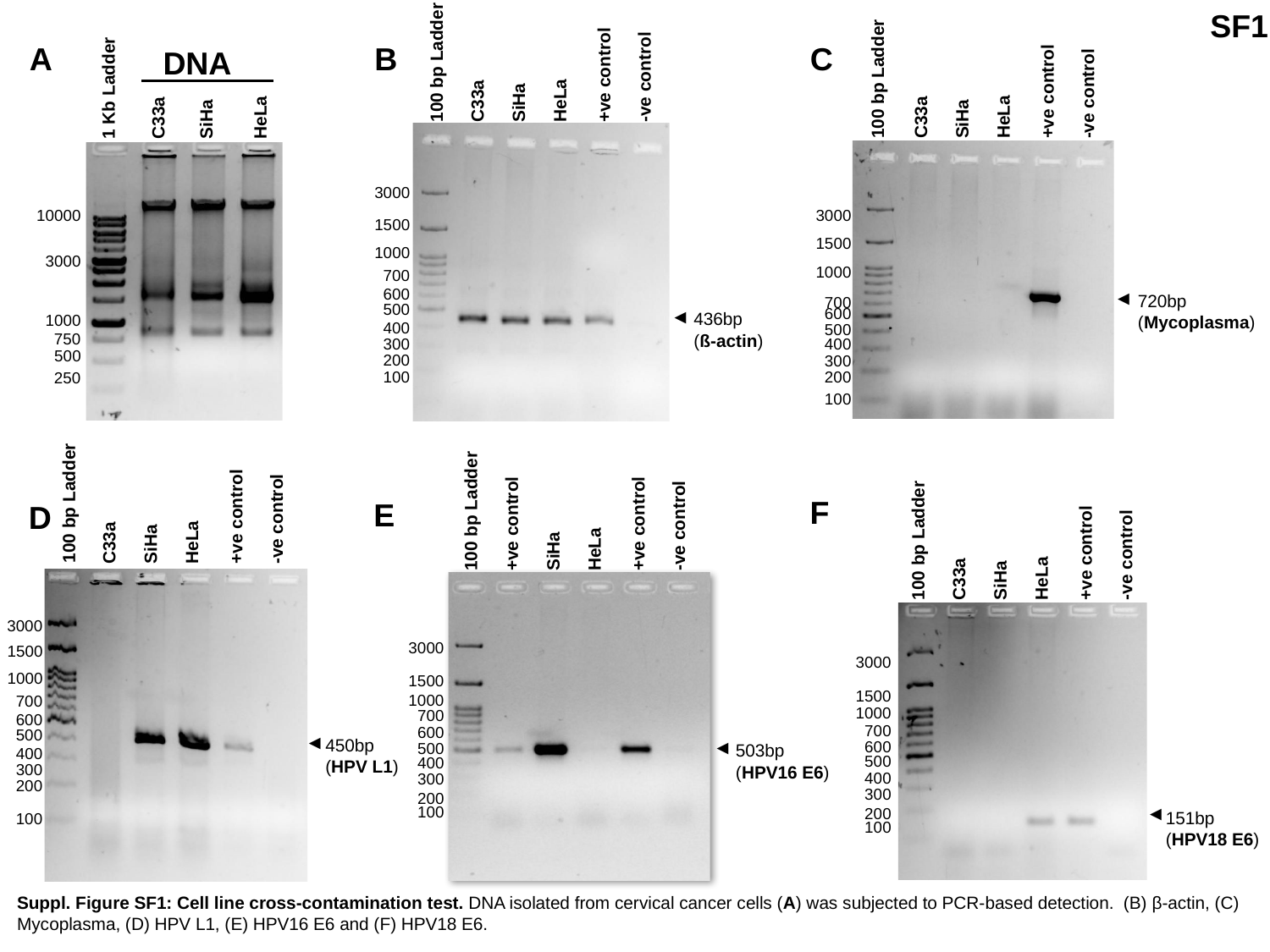

SF1
A
B
C
DNA
100 bp Ladder
-ve control
+ve control
SiHa
100 bp Ladder
+ve control
-ve control
C33a
1 Kb Ladder
HeLa
SiHa
HeLa
HeLa
C33a
SiHa
C33a
3000
10000
3000
1500
1500
1000
3000
1000
700
600
►
720bp
(Mycoplasma)
700
500
600
►
436bp
(ß-actin)
1000
400
500
750
300
400
500
200
300
200
100
250
100
100 bp Ladder
100 bp Ladder
F
E
D
+ve control
+ve control
+ve control
SiHa
-ve control
HeLa
C33a
-ve control
100 bp Ladder
SiHa
HeLa
-ve control
+ve control
C33a
SiHa
HeLa
3000
3000
1500
3000
1000
1500
1500
1000
700
1000
700
600
700
600
500
►
450bp
(HPV L1)
600
500
►
503bp
(HPV16 E6)
400
500
400
300
400
300
200
300
200
100
200
►
151bp
(HPV18 E6)
100
100
Suppl. Figure SF1: Cell line cross-contamination test. DNA isolated from cervical cancer cells (A) was subjected to PCR-based detection. (B) β-actin, (C) Mycoplasma, (D) HPV L1, (E) HPV16 E6 and (F) HPV18 E6.

## Slide 2
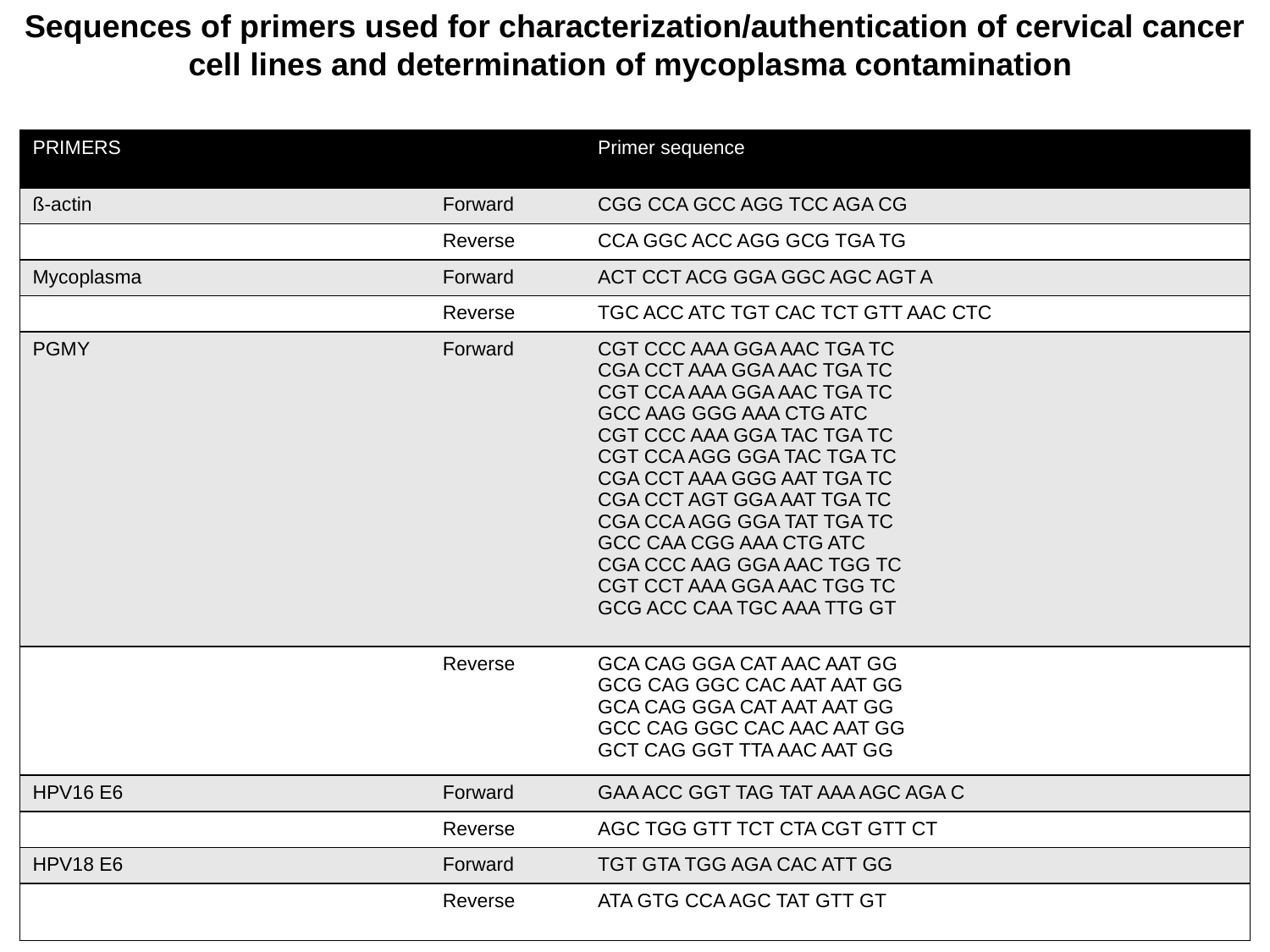

Sequences of primers used for characterization/authentication of cervical cancer cell lines and determination of mycoplasma contamination
| PRIMERS | | Primer sequence |
| --- | --- | --- |
| ß-actin | Forward | CGG CCA GCC AGG TCC AGA CG |
| | Reverse | CCA GGC ACC AGG GCG TGA TG |
| Mycoplasma | Forward | ACT CCT ACG GGA GGC AGC AGT A |
| | Reverse | TGC ACC ATC TGT CAC TCT GTT AAC CTC |
| PGMY | Forward | CGT CCC AAA GGA AAC TGA TC CGA CCT AAA GGA AAC TGA TC CGT CCA AAA GGA AAC TGA TC GCC AAG GGG AAA CTG ATC CGT CCC AAA GGA TAC TGA TC CGT CCA AGG GGA TAC TGA TC CGA CCT AAA GGG AAT TGA TC CGA CCT AGT GGA AAT TGA TC CGA CCA AGG GGA TAT TGA TC GCC CAA CGG AAA CTG ATC CGA CCC AAG GGA AAC TGG TC CGT CCT AAA GGA AAC TGG TC GCG ACC CAA TGC AAA TTG GT |
| | Reverse | GCA CAG GGA CAT AAC AAT GG GCG CAG GGC CAC AAT AAT GG GCA CAG GGA CAT AAT AAT GG GCC CAG GGC CAC AAC AAT GG GCT CAG GGT TTA AAC AAT GG |
| HPV16 E6 | Forward | GAA ACC GGT TAG TAT AAA AGC AGA C |
| | Reverse | AGC TGG GTT TCT CTA CGT GTT CT |
| HPV18 E6 | Forward | TGT GTA TGG AGA CAC ATT GG |
| | Reverse | ATA GTG CCA AGC TAT GTT GT |

## Slide 3
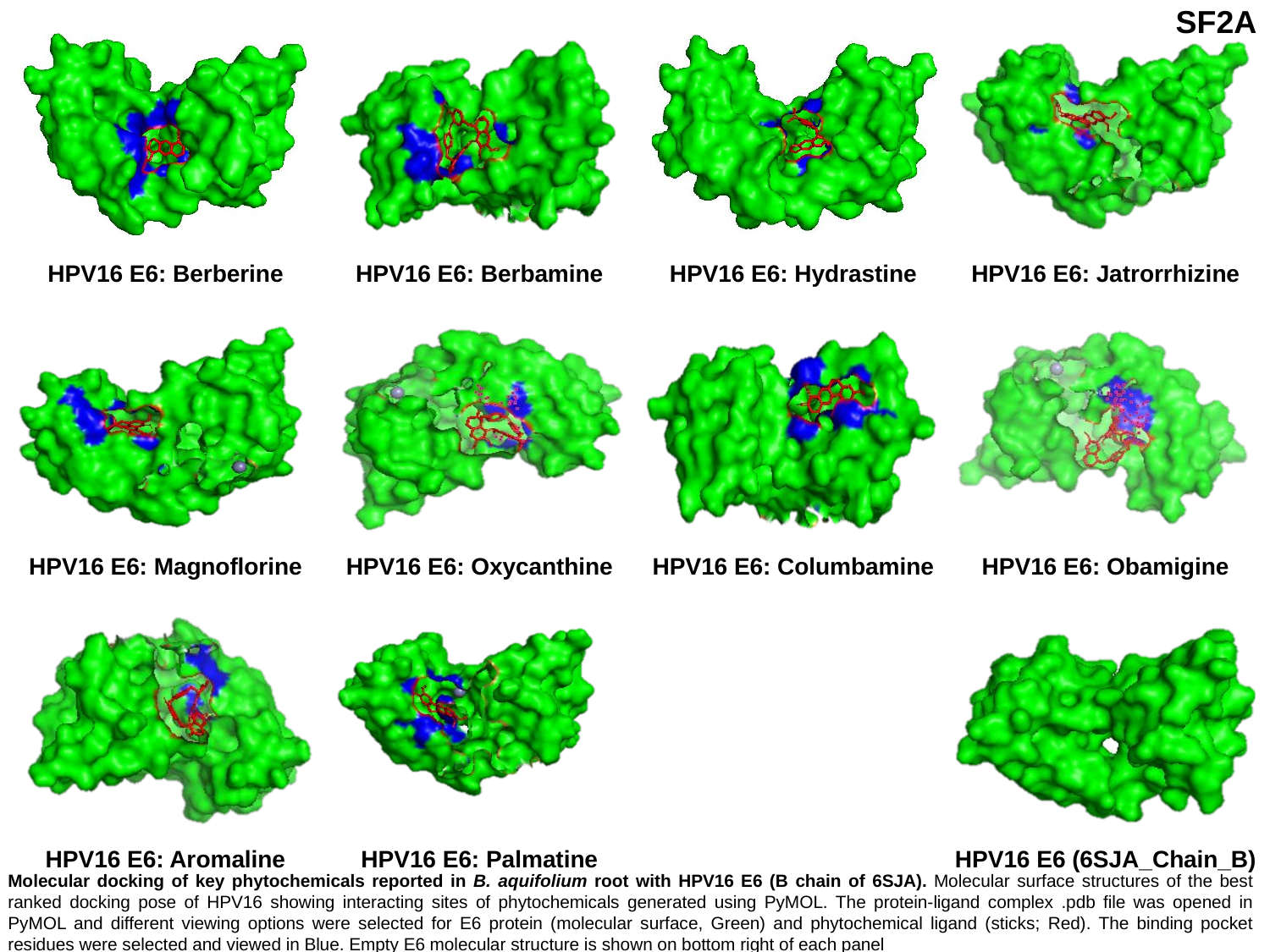

SF2A
HPV16 E6: Berberine
HPV16 E6: Berbamine
HPV16 E6: Hydrastine
HPV16 E6: Jatrorrhizine
HPV16 E6: Magnoflorine
HPV16 E6: Oxycanthine
HPV16 E6: Columbamine
HPV16 E6: Obamigine
HPV16 E6: Aromaline
HPV16 E6: Palmatine
HPV16 E6 (6SJA_Chain_B)
Molecular docking of key phytochemicals reported in B. aquifolium root with HPV16 E6 (B chain of 6SJA). Molecular surface structures of the best ranked docking pose of HPV16 showing interacting sites of phytochemicals generated using PyMOL. The protein-ligand complex .pdb file was opened in PyMOL and different viewing options were selected for E6 protein (molecular surface, Green) and phytochemical ligand (sticks; Red). The binding pocket residues were selected and viewed in Blue. Empty E6 molecular structure is shown on bottom right of each panel

## Slide 4
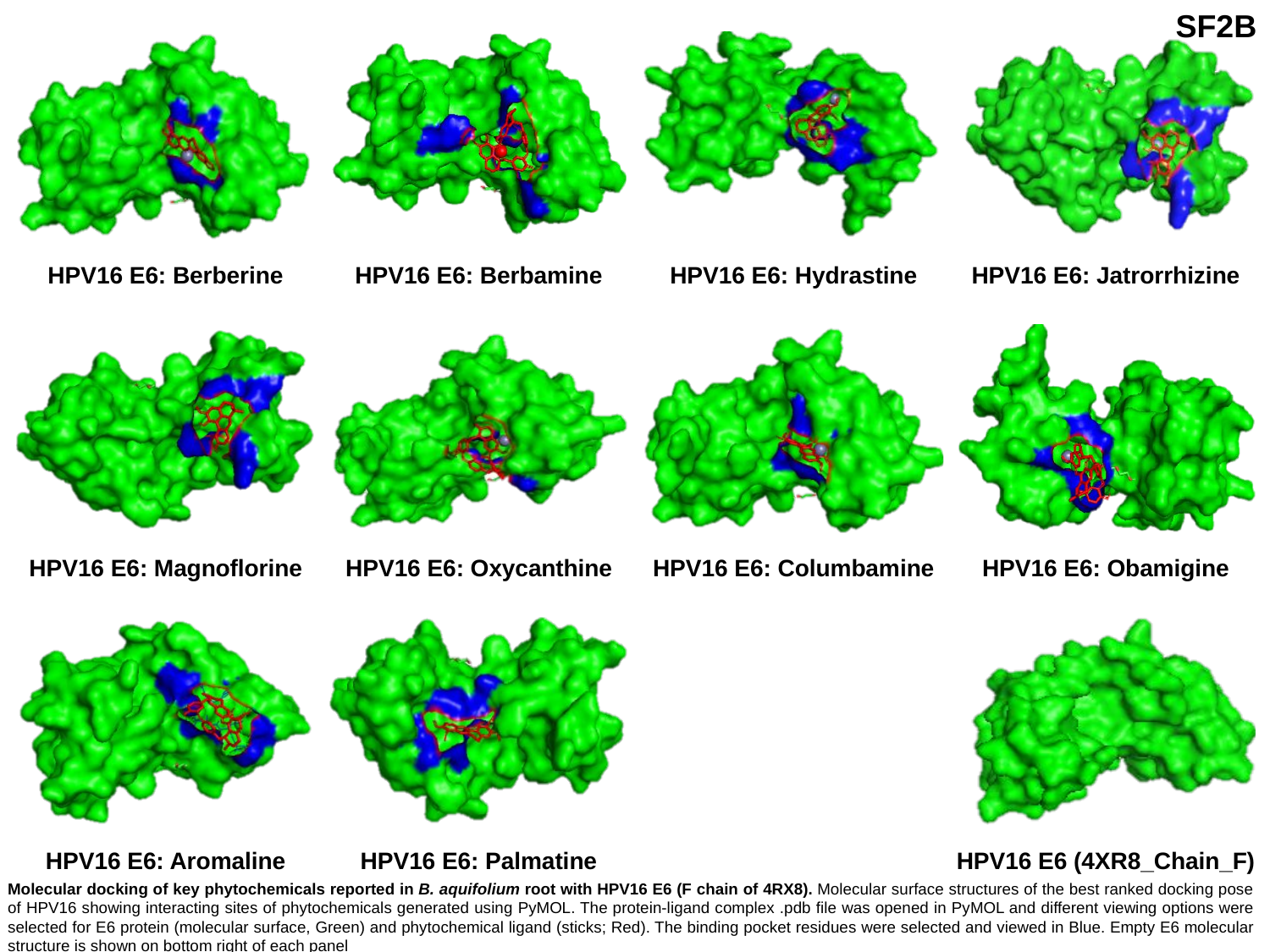

SF2B
HPV16 E6: Berberine
HPV16 E6: Berbamine
HPV16 E6: Hydrastine
HPV16 E6: Jatrorrhizine
HPV16 E6: Magnoflorine
HPV16 E6: Oxycanthine
HPV16 E6: Columbamine
HPV16 E6: Obamigine
HPV16 E6: Aromaline
HPV16 E6: Palmatine
HPV16 E6 (4XR8_Chain_F)
Molecular docking of key phytochemicals reported in B. aquifolium root with HPV16 E6 (F chain of 4RX8). Molecular surface structures of the best ranked docking pose of HPV16 showing interacting sites of phytochemicals generated using PyMOL. The protein-ligand complex .pdb file was opened in PyMOL and different viewing options were selected for E6 protein (molecular surface, Green) and phytochemical ligand (sticks; Red). The binding pocket residues were selected and viewed in Blue. Empty E6 molecular structure is shown on bottom right of each panel
