# Supplementary material for: Targeting Aberrant Expression of STAT3 and AP-1 Oncogenic Transcription Factors and HPV Oncoproteins in Cervical Cancer by Berberis aquifolium
Source: Front Pharmacol. 2021 Oct 28;12:757414. doi: 10.3389/fphar.2021.757414 (PMC8580881; doi:10.3389/fphar.2021.757414)
Supplement: Supplementary file 3 [file Table2.DOCX]

**Supplementary Table 2: HPV16 E6 amino acid residues involved in interaction with respective cellular targets.**

| **S. No.** | **Cellular Target/Interacting Partners of E6** | **Interacting E6 Residues** | | **Reference** |
| --- | --- | --- | --- | --- |
|  |  | **Positions reported** | **Position in UNIPROT SEQ P03126** |  |
| **1** | p53 | Q6, E7, R8, R10, Q14, E18, Y43, D44, F47, D49, L100, P112 | Q13, E14, R15, R17, Q21, E25, Y50, D51, F54, D56, L107, P119 | **(Martinez-Zapien *et al.* 2016)** |
| **2** | E6AP | R10, K11, C51, R55, S74, R77, H78, R102, R129, R131 | R17, K18, C58, R62, S81, R84, H85, R109, R136, R138 | **(Zanier *et al.* 2013)** |
| **3** | PDZ Domain containing proteins (MAGI1, hDlg1, CFTR Associated Ligand etc.) | R148, S149, S150, R151, T152, R153, R154, E155, T156, Q157 | R148, S149, S150, R151, T152, R153, R154, E155, T156, Q157 | **(Thomas *et al.* 2008)** |
| **4** | Zn | C33, C63, C66, C103, C106, C136 | C40, C70, C73, C110, C113, C143 | **(Kanda *et al.* 1991)** |

**References:**

**Kanda, T., Watanabe, S., Zanma, S., Sato, H., Furuno, A. and Yoshiike, K. (1991)**. Human papillomavirus type 16 E6 proteins with glycine substitution for cysteine in the metal-binding motif. Virology **185**(2): 536-43.

**Martinez-Zapien, D., Ruiz, F. X., Poirson, J., Mitschler, A., Ramirez, J., Forster, A., Cousido-Siah, A., Masson, M., Vande Pol, S., Podjarny, A., Trave, G. and Zanier, K. (2016)**. Structure of the E6/E6AP/p53 complex required for HPV-mediated degradation of p53. Nature **529**(7587): 541-5.

**Thomas, M., Dasgupta, J., Zhang, Y., Chen, X. and Banks, L. (2008)**. Analysis of specificity determinants in the interactions of different HPV E6 proteins with their PDZ domain-containing substrates. Virology **376**(2): 371-8.

**Zanier, K., Charbonnier, S., Sidi, A. O., McEwen, A. G., Ferrario, M. G., Poussin-Courmontagne, P., Cura, V., Brimer, N., Babah, K. O., Ansari, T., Muller, I., Stote, R. H., Cavarelli, J., Vande Pol, S. and Trave, G. (2013)**. Structural basis for hijacking of cellular LxxLL motifs by papillomavirus E6 oncoproteins. Science **339**(6120): 694-8.
