# Supplementary material for: Targeting Aberrant Expression of STAT3 and AP-1 Oncogenic Transcription Factors and HPV Oncoproteins in Cervical Cancer by Berberis aquifolium
Source: Front Pharmacol. 2021 Oct 28;12:757414. doi: 10.3389/fphar.2021.757414 (PMC8580881; doi:10.3389/fphar.2021.757414)
Supplement: Supplementary file 4 [file Table3.DOCX]

**Supplementary Table ST3: List of top 10 conformations generated in molecular docking of phytochemicals reported in *B. aquifolium* root using HPV16 E6 crystal structure 6JSA (Chain B) along with the corresponding binding energies (B.E. in kcal/mol) and inhibition constants (I.C. in µM).**

| **S. No.** | **Ligand** | **No.** |  | **Conformations** | | | | | | | | | | |
| --- | --- | --- | --- | --- | --- | --- | --- | --- | --- | --- | --- | --- | --- | --- |
|  |  |  |  | **1** | **2** | **3** | **4** | **5** | **6** | **7** | **8** | **9** | **10** |  |
| **1** | **Aromaline** | **3** | **B.E.** | **-6.16** | **-5.9** |  |  |  |  |  |  |  |  |  |
|  |  |  | I.C. | 30.5 | 47.09 |  |  |  |  |  |  |  |  |  |
| **2** | **Berbamine** | **10** | **B.E.** | **-6.14** | **-5.93** | **-5.89** | **-5.65** | **-5.56** | **-5.35** | **-5.12** | **-4.8** | **-4.65** | **-4.32** |  |
|  |  |  | I.C. | 31.69 | 45.12 | 47.87 | 72.73 | 84.59 | 119.39 | 176.21 | 303.63 | 392.13 | 679.26 |  |
| **3** | **Berberine** | **10** | **B.E.** | **-75800000** | **-74600000** | **-69400000** | **-68900000** | **-68800000** | **-67700000** | **-6.63** | **-6.59** | **-6.59** | **-6.47** |  |
|  |  |  | I.C. | 0.0 | 0.0 | 0.0 | 0.0 | 0.0 | 0.0 | 13.7 | 14.75 | 14.75 | 18.1 |  |
| **4** | **Columbamine** | **10** | **B.E.** | **-5.0** | **-5.0** | **-4.72** | **-4.67** | **-4.66** | **-4.59** | **-4.5** | **-4.46** | **-4.37** | **-4.23** |  |
|  |  |  | I.C. | 214.66 | 215.36 | 344.93 | 376.07 | 383.29 | 430.45 | 506.65 | 538.01 | 623.33 | 789.81 |  |
| **5** | **Hydrastine** | **10** | **B.E.** | **-75600000** | **-75500000** | **-75100000** | **-69400000** | **-69200000** | **-69000000** | **-69000000** | **-68600000** | **-4.53** | **-3.61** |  |
|  |  |  | I.C. | 0.0 | 0.0 | 0.0 | 0.0 | 0.0 | 0.0 | 0.0 | 0.0 | 476.27 | 2240 |  |
| **6** | **Jatrorrhizine** | **10** | **B.E.** | **-5.99** | **-5.98** | **-5.92** | **-5.81** | **-5.69** | **-5.57** | **-5.43** | **-5.39** | **-5.36** | **-4.94** |  |
|  |  |  | I.C. | 40.92 | 41.1 | 45.96 | 55.49 | 67.0 | 83.19 | 104.36 | 111.23 | 118.69 | 240.0 |  |
| **7** | **Magnoflorine** | **10** | **B.E.** | **-7.08** | **-7.07** | **-6.09** | **-5.8** | **-5.6** | **-5.54** | **-5.43** | **-5.27** | **-4.62** | **-4.31** |  |
|  |  |  | I.C. | 6.46 | 6.62 | 34.11 | 56.02 | 78.66 | 87.42 | 103.88 | 137.62 | 409.94 | 687.56 |  |
| **8** | **Obamigine** | **10** | **B.E.** | **-5.56** | **-5.5** | **-5.41** | **-4.74** | **-4.74** | **-4.65** | **-4.65** | **-4.45** | **-4.36** | **-4.06** |  |
|  |  |  | I.C. | 83.87 | 92.85 | 107.99 | 333.89 | 335.1 | 392.22 | 393.56 | 547.66 | 634.21 | 1.06mM |  |
| **9** | **Oxyacanthine** | **10** | **B.E.** | **-6.05** | **-5.96** | **-5.91** | **-5.68** | **-5.42** | **-5.34** | **-5.26** | **-5.24** | **-5.07** | **-4.69** |  |
|  |  |  | I.C. | 36.73 | 42.96 | 46.76 | 69.1 | 105.78 | 122.07 | 138.76 | 144.13 | 191.86 | 365.89 |  |
| **10** | **Palmatine** | **10** | **B.E.** | **-76000000** | **-75900000** | **-75900000** | **-75700000** | **-75400000** | **-74200000** | **-74100000** | **-73600000** | **-72200000** | **-6.69** |  |
|  |  |  | I.C. | 0.0 | 0.0 | 0.0 | 0.0 | 0.0 | 0.0 | 0.0 | 0.0 | 0.0 | 12.55 |  |

**Supplementary Table ST4: List of top 10 conformations generated in molecular docking of phytochemicals reported in *B. aquifolium* root using HPV16 E6 crystal structure 4XR8 (Chain F) along with the corresponding binding energies (B.E. in kcal/mol) and inhibition constants (I.C. in µM).**

| **S. No.** | **Ligand** | **No.** |  | **Conformations** | | | | | | | | | |
| --- | --- | --- | --- | --- | --- | --- | --- | --- | --- | --- | --- | --- | --- |
|  |  |  |  | **1** | **2** | **3** | **4** | **5** | **6** | **7** | **8** | **9** | **10** |
| **1** | **Aromaline** | **3** | **B.E.** | **-4.08** | **-4.0** | **-3.44** |  |  |  |  |  |  |  |
|  |  |  | I.C. | 1020 | 1170 | 2990 |  |  |  |  |  |  |  |
| **2** | **Berbamine** | **10** | **B.E.** | **-3.87** | **-3.59** | **-3.49** | **-3.23** | **-3.16** | **-3.02** | **-2.87** | **-2.86** | **-2.55** | **-2.29** |
|  |  |  | I.C. | 1460 | 2350 | 2750 | 4280 | 4860 | 6150 | 7920 | 8050 | 13550 | 21060 |
| **3** | **Berberine** | **10** | **B.E.** | **-73400000** | **-5.07** | **-4.78** | **-4.76** | **-4.64** | **-4.63** | **-4.58** | **-4.56** | **-4.44** | **-3.92** |
|  |  |  | I.C. | 0.0 | 193.36 | 314.65 | 323.33 | 398.46 | 405.25 | 441.52 | 455.89 | 560.1 |  |
| **4** | **Columbamine** | **10** | **B.E.** | **-5.24** | **-4.85** | **-4.52** | **-4.47** | **-4.06** | **-4.02** | **-3.74** | **-3.7** | **-3.52** | **-3.23** |
|  |  |  | I.C. | 144.25 | 277.65 | 483.59 | 526.95 | 1050 | 1140 | 1800 | 1930 | 2610 | 4270 |
| **5** | **Hydrastine** | **10** | **B.E.** | **-68900000** | **-62000000** | **-3.98** | **-3.86** | **-3.82** | **-3.51** | **-3.46** | **-3.4** | **-3.05** | **-2.95** |
|  |  |  | I.C. | 0.0 | 0.0 | 1220 | 1480 | 1580 | 2670 | 2930 | 3220 | 5770 | 6850 |
| **6** | **Jatrorrhizine** | **10** | **B.E.** | **-5.4** | **-4.83** | **-4.4** | **-4.34** | **-4.25** | **-4.1** | **-3.94** | **-3.86** | **-3.72** | **415000** |
|  |  |  | I.C. | 110.2 | 288.0 | 600.06 | 660.83 | 765.93 | 984.45 | 1290 | 1480 | 1870 | 415000 |
| **7** | **Magnoflorine** | **10** | **B.E.** | **-5.58** | **-5.0** | **-4.48** | **-4.3** | **-4.01** | **-3.79** | **-3.79** | **-3.71** | **-3.6** | **-3.31** |
|  |  |  | I.C. | 80.94 | 216.11 | 517.39 | 708.17 | 1160 | 1660 | 1670 | 1900 | 2310 | 3720 |
| **8** | **Obamigine** | **10** | **B.E.** | **-3.51e+024** | **-4.2** | **-3.98** | **-3.73** | **-3.69** | **-3.68** | **-3.67** | **-3.42** | **-2.79** | **-2.71** |
|  |  |  | I.C. | 0.0 | 831.34 | 1210 | 1840 | 1990 | 1990 | 2030 | 3110 | 8960 | 10360 |
| **9** | **Oxyacanthine** | **10** | **B.E.** | **-3.85** | **-3.46** | **-3.46** | **-3.11** | **-2.88** | **-2.86** | **-2.81** | **-2.79** | **-2.57** | **-2.35** |
|  |  |  | I.C. | 1500 | 2900 | 2890 | 5230 | 7740 | 7980 | 8650 | 8960 | 13110 | 18960 |
| **10** | **Palmatine** | **10** | **B.E.** | **-75700000** | **-5.7** | **-4.66** | **-4.51** | **-4.41** | **-4.22** | **-3.88** | **-3.46** | **-3.29** | **-3.27** |
|  |  |  | I.C. | 0.0 | 66.37 | 381.13 | 494.99 | 583.57 | 800.99 | 1430 | 2930 | 3890 | 4040 |
